# Early antiviral treatment following gammaherpesvirus-68 infection of the central nervous system prevents subsequent multiple sclerosis-like disease

**DOI:** 10.1186/s12974-025-03547-8

**Published:** 2025-10-08

**Authors:** Alexander Muselman, Sameera Kongara, Nathan Hsu, Asha Aggarwal, Joanna Yu, Jayakumar Rajadas, Edgar G. Engleman

**Affiliations:** 1 Department of Pathology, Stanford University, Stanford, CA 94305 USA; 2 Advanced Drug Delivery and Regenerative Biomaterials Laboratory, Cardiovascular Institute, Department of Medicine, School of Medicine, Stanford University, Palo Alto, CA USA; 3 Department of Bioengineering and Therapeutic Sciences, School of Pharmacy, University of California, San Francisco, CA USA; 4 Stanford Cancer Institute, Stanford University, Palo Alto, CA 94305 USA

**Keywords:** Multiple sclerosis, Epstein-Barr virus, Microglia, Immune priming, Neuroinflammation, Metabolism, Iron

## Abstract

**Background:**

Growing evidence indicates that Epstein-Barr virus (EBV), a gammaherpesvirus, plays a central role in the pathogenesis of multiple sclerosis (MS). The presence of EBV-infected cells in the central nervous system (CNS) of MS patients, but not in neurologically healthy individuals, suggests that viral persistence in the CNS may drive MS. However, why there is such a long interval between initial infection and the development of disease is unknown.

**Methods:**

To model the effects of EBV infection on the brain, we intracerebrally infected mice with murine gammaherpesvirus-68 (MHV68), a virus genetically related to EBV that causes transient pathology strikingly similar to that seen in humans after acute EBV infection. One month following MHV68 infection, we administered myelin oligodendrocyte glycoprotein (MOG) peptide to evaluate the effects of prior MHV68 infection on the response to an additional inflammatory stimulus of the CNS. Virus persistence, microglial activation and immune cell infiltration were evaluated over time using flow cytometry.

**Results:**

Intracerebral MHV68 infection induced mild brain demyelination and ataxia, a common symptom of MS, that both quickly resolved. However, administration of MOG peptide one month later led to more severe brain demyelination and more sustained ataxia, suggesting that prior MHV68 infection sensitized the mice to a newly introduced immune stimulus. Further investigation revealed that following CNS infection, MHV68 persisted in microglia, where it induced a primed phenotype marked by elevated MHC-II expression and heightened immune reactivity for at least six months. Primed microglia displayed increases in the labile iron pool, and iron chelation reduced microglial priming. Early antiviral treatment during MHV68 infection completely prevented subsequent MOG-induced demyelinating disease.

**Conclusions:**

These findings support a two-step mechanism by which CNS infection with a gammaherpesvirus closely related to EBV sensitizes the host to a second unrelated immune stimulus that triggers MS-like disease manifestations. Chronic priming of microglia resulting from the initial infection contributes to this process, and prevention of such priming with early antiviral treatment also prevents neuropathology following the second stimulus. EBV infection may similarly sensitize humans to a second stimulus and, if so, treatment of acute EBV infection may avert subsequent MS development.

**Supplementary Information:**

The online version contains supplementary material available at 10.1186/s12974-025-03547-8.

## Background

Multiple sclerosis (MS) is a chronic neuroinflammatory disease characterized by focal demyelination and diffuse tissue injury in the brain and spinal cord [1–4]. Although the precise cause of MS remains unclear, a preponderance of evidence points to Epstein-Barr virus (EBV), a gammaherpesvirus responsible for infectious mononucleosis in adolescents and young adults, as a key component in its development [5–7]. The mechanisms underlying EBV’s contribution to MS pathogenesis are complex and likely multifactorial. MS could arise from impaired EBV-specific immune control [8], EBV-specific antibodies that cross-react with central nervous system (CNS) autoantigens [9–14], persistent activation of autoreactive B and T cells [15–17], or uncontrolled EBV infection [18, 19]. EBV can establish latency in various cell types, including B cells and macrophages [20–22], where it induces alterations in their proliferation, transcriptional profile, and metabolism [23, 24]–processes that may also contribute to MS pathogenesis. Furthermore, under certain conditions, EBV can reactivate, leading to the release of viral particles through host cell death and facilitating viral spread within the local microenvironment [25, 26].

The detection of EBV gene and protein expression specifically in the brains of MS patients, but not in neurologically healthy individuals, suggests that persistent EBV infection, in either the latent or the lytic phase, within the CNS may contribute to MS [27–30]. This hypothesis is further supported by observations from viral mouse models of MS, where intracerebral inoculation with viruses such as Theiler’s murine encephalomyelitis virus (TMEV), murine hepatitis virus (MHV), and Semliki Forest virus (SFV) induces chronic inflammation and demyelination in the spinal cord, driven by viral persistence in microglia, macrophages, and astrocytes [31–34]. However, these models employ single-stranded RNA viruses not typically associated with MS, raising questions about whether a gammaherpesvirus-like EBV could induce similar demyelinating disease. Interestingly, the identification of simian herpesvirus in lesions from spontaneous encephalomyelitis in a Japanese macaque colony suggests that gammaherpesviruses may contribute to similar pathologies in humans [35].

In addition to B cells, EBV gene and protein expression have been detected in microglia, the primary resident immune cells of the CNS [29, 30]. Microglia are thought to be pivotal in the pathogenesis of MS and its mouse model, experimental autoimmune encephalomyelitis (EAE), due to their involvement in antigen presentation, myelin debris clearance, and modulation of other immune and glial cells [36–38]. Microglial nodules, where HLA-DR + microglia accumulate and cluster in the normal-appearing white matter (NAWM), are thought to precede MS lesion formation and are associated with more severe MS pathology [39–41]. Although prior studies have focused on microglia in fully developed lesions [36, 37], the mechanisms driving early microglial dysfunction remain poorly understood.

We hypothesized that gammaherpesvirus infection of the brain may lead to infection of microglia that might have a long-term impact on the brain. To test this hypothesis, in the current study we infected mice intracerebrally with murine gammaherpesvirus-68 (MHV68), a naturally occurring pathogen in mice that has extensive genetic and phenotypic similarities to EBV and induces pathology, including marked splenomegaly and lymphoproliferative diseases, strikingly similar to that seen in humans after acute EBV infection [42]. Because the midbrain serves as a functional bridge between the brain and spinal cord [43], we targeted this region to model how virus-induced neuroinflammation in a central region that integrates motor, sensory, and autonomic pathways can contribute to the heterogeneous pathology of MS. Our results show that following intracerebral MHV68 infection, the virus persisted in microglia, resulting in microglial priming and altered metabolism. Notably, mice with sustained microglial infection that were subsequently injected with myelin oligodendrocyte glycoprotein (MOG) peptide developed midbrain demyelination and exacerbated ataxia, a lack of coordination of voluntary muscle movement [44, 45]. Treatment with an anti-viral agent, cidofovir, immediately after infection prevented MOG peptide-induced ataxia. These findings suggest that persistent gammaherpesvirus infection in microglia increases susceptibility to demyelination following subsequent exposure to inflammatory stimuli.

## Results

### Intracerebral MHV68 infection spreads minimally to the spinal cord but predisposes mice to exacerbated demyelination in the brain and ataxia following MOG peptide administration

**Fig. 1:**
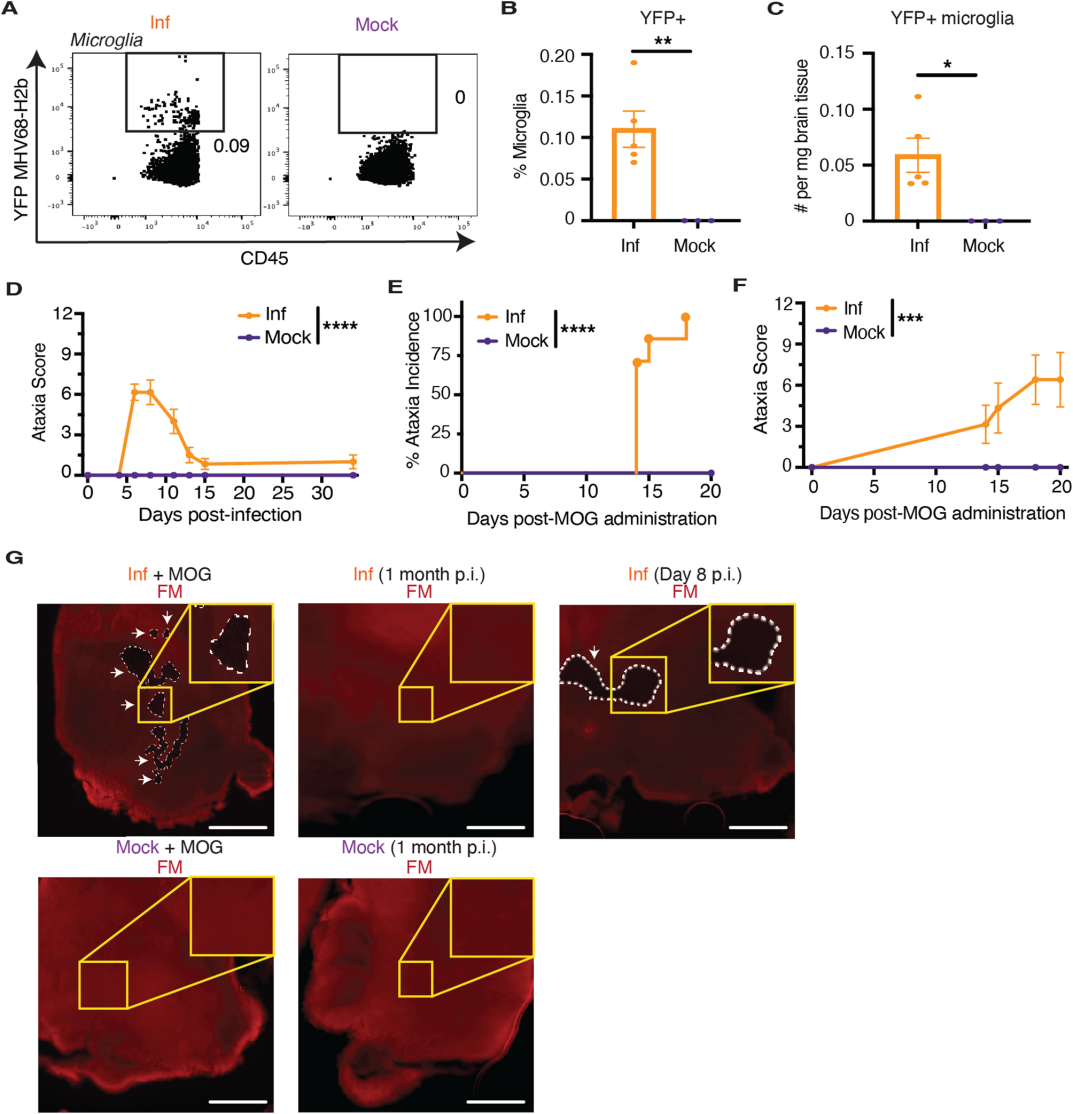
Intracerebral MHV68 infection predisposes mice to brain demyelination and ataxia following MOG peptide administration. **A** Brains were isolated on day 32 after intracerebral MHV68-H2bYFP (Inf, *n* = 5) or mock (*n* = 3) infection, and the (**B**) frequency and (**C**) absolute number of MHV68-H2bYFP + microglia were determined. **D** Ataxia score following intracerebral MHV68 (Inf, *n* = 10) or mock (*n* = 4) infection. **E** Incidence and (**F**) severity of ataxia following MOG peptide administration in mice with persistent intracerebral MHV68 (Inf, *n* = 7) or mock-infected (*n* = 10). **G** Brains were isolated in MHV68-infected (Inf) or mock-infected mice one month or 8 days post-infection (p.i.) or 15 days after MOG peptide administration. Fixed frozen sections were stained with FluoroMyelin Red (FM) to assess demyelination. Demyelinated lesion borders are marked with dashed line and arrows. Scale bar, 1000 μm. Statistical significance was determined using an unpaired two-tailed *t* test in **B** and **C**, a mixed-effects model in **D** and **F**, and a log-rank test in **E**. Data are represented as mean ± standard error of mean (SEM). * *p* < 0.05, ** *p* < 0.01, *** *p* < 0.001, and **** *p* < 0.0001

Previous studies have demonstrated that MHV68 can persist in the brain following intracerebral infection [46, 47], but it is unclear whether the virus also spreads to the spinal cord, as seen with intracerebral infection with non-gammaherpesviruses like TMEV [31]. To investigate this question, we intracerebrally infected mice with a transgenic MHV68 carrying an EYFP-histone H2b fusion gene (MHV68-H2bYFP) that enables direct detection of persistent CNS infection, including both latent and lytic phases, based on YFP expression [48]. One month post-infection, we observed more YFP-expressing cells in the brain compared to the spinal cord (Additional File 1: Fig. S1, A-C), suggesting persistence of the virus in the brain but limited viral spread to the spinal cord. Among the YFP-expressing cells were microglia (Fig. 1, A-C), oligodendrocytes (Additional File 1: Fig. S1, D-F), and astrocytes (Additional File 1: Fig. S1, G-I). Although infected mice showed mild gait disturbances one week post-infection, these symptoms quickly resolved and the mice did not develop the chronic hindlimb paralysis associated with TMEV-induced demyelinating disease (TMEV-IDD) (Additional File 1: Fig. S2A). These findings indicate that intracerebral MVH68 infection alone is insufficient to induce pathology similar to TMEV-IDD, likely due to minimal viral dissemination to the spinal cord.

Previous studies have shown that mice infected with MHV68 intraperitoneally exhibited heightened sensitivity to myelin oligodendrocyte glycoprotein (MOG) peptide-induced EAE, with neuropathology more closely resembling that of MS than when EAE is induced by MOG peptide alone [49, 50]. To determine whether the same stimulus could drive demyelinating disease in mice with persistent intracerebral MHV68 infection, we administered MOG peptide to mice one month post-infection. MOG peptide-challenged, MHV68-infected mice exhibited delayed onset and milder classical EAE, also characterized by hindlimb paralysis, compared to mock-infected mice (Additional File 1: Fig. S2, B and C). Previously infected mice challenged with MOG peptide also did not exhibit demyelinated lesions in the spinal cord, in contrast to mock-infected mice (Additional File 1: Fig. S2D). Next, due to the connection between the midbrain and the cerebellum, we assessed the effect of intracerebral MHV68 infection on cerebellar ataxia, a hallmark of brain inflammation observed acutely in some individuals following acute EBV infection. In the absence of a second stimulus, the infected mice developed ataxia that resolved approximately one week post-infection (Fig. 1D). However, upon challenge with MOG peptide, the previously infected mice displayed a more severe and persistent ataxia compared to that seen with MHV68 infection alone (Fig. 1, E and F). Only MHV68-infected mice exhibited demyelinated lesions in the midbrain following challenge with MOG peptide (Fig. 1G). Injection of lipopolysaccharide (LPS) into the midbrain also resulted in larger lesions in previously infected mice than in mock-infected mice (Additional File 1: Fig. S3). Together, these findings suggest that viral persistence in brain may prime mice for brain-targeted inflammation and demyelination more akin to MS following exposure to a second immune stimulus.

### Persistent intracerebral MHV68 infection drives long-term expansion and priming of microglia

**Fig. 2:**
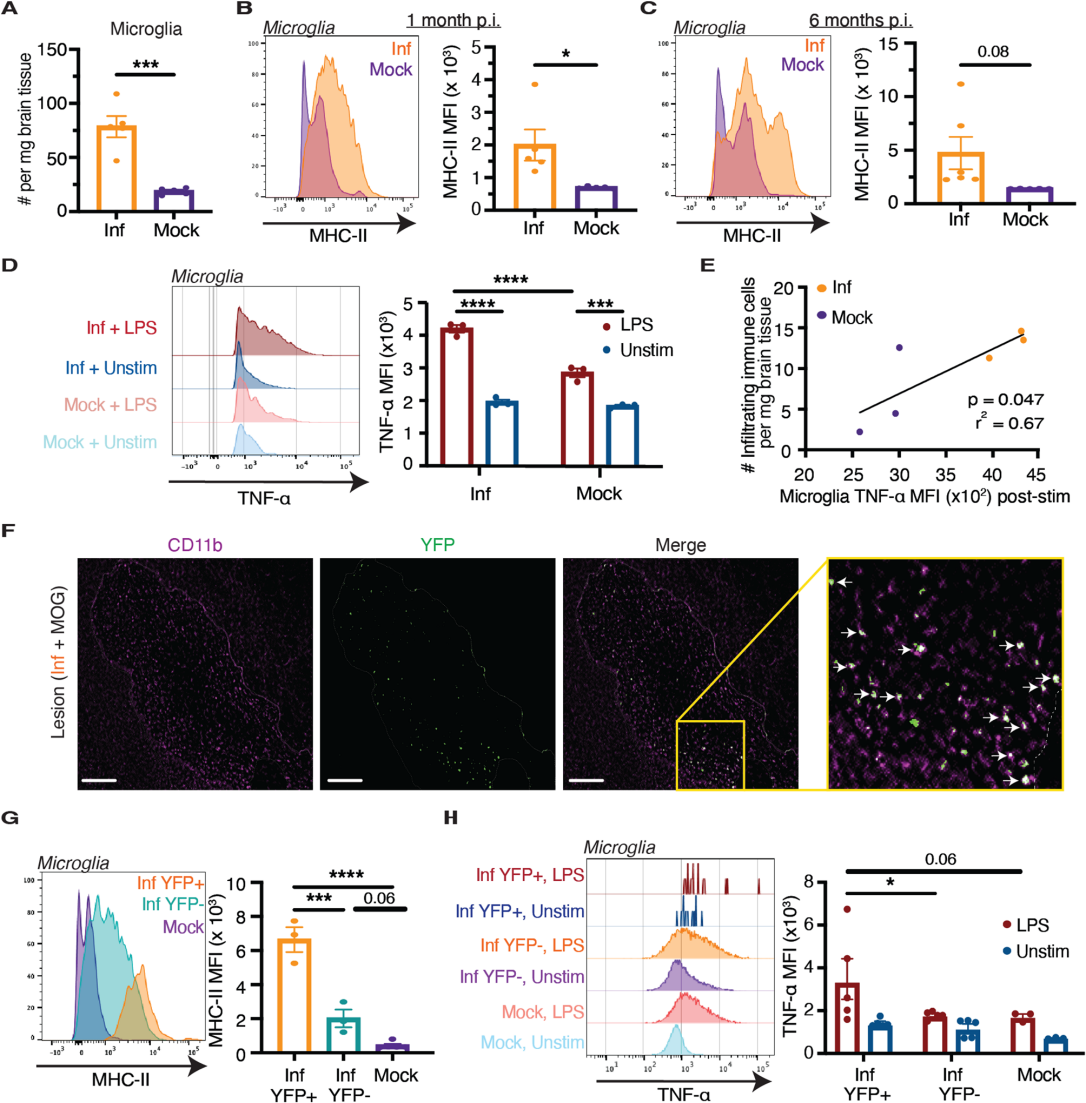
Persistent intracerebral MHV68 infection drives long-term expansion and priming of microglia. **A** Absolute number of microglia in mice on day 30 following intracerebral MHV68 (Inf, *n* = 5) or mock infection (*n* = 5). MHC-II expression on microglia (**B**) one month and (**C**) six months following intracerebral MHV68 (Inf, *n* = 5) or mock (*n* = 5) infection. **D** Intracellular TNF-α expression, at baseline and upon stimulation with LPS, in microglia isolated from mice on day 30 following intracerebral MHV68 (Inf, *n* = 3) or mock infection (*n* = 3). **E** Correlation between microglial TNF-α expression post-stimulation and the absolute number of brain-infiltrating immune cells in mice on day 30 after intracerebral MHV68 (Inf) or mock infection. **F** CD11b and MHV68-H2bYFP staining in a demyelinated lesion from the brain of a MHV68H2bYFP-infected mouse 15 days after MOG peptide administration. Demyelinated lesion borders are marked with dashed line, and YFP + microglia are marked with arrows. Scale bar, 50 μm. **G** MHC-II expression among YFP + and YFP- microglia on day 30 following intracerebral MHV68-H2bYFP (Inf, *n* = 3) or mock infection (*n* = 5). **H** Intracellular TNF-α expression, at baseline and upon stimulation with LPS, in YFP + and YFP- microglia on day 30 following intracerebral MHV68-H2bYFP (Inf, *n* = 5) or mock infection (*n* = 3). Statistical significance was determined using an unpaired two-tailed *t* test in **A**, **B**, and **C**, a two-way ANOVA with Šidák multiple comparisons in **D** and **H**, a simple linear regression in **E**, and an ordinary one-way ANOVA with Tukey’s correction for multiple comparisons in **G**. Data are represented as mean ± standard error of mean (SEM). * *p* < 0.05, ** *p* < 0.01, *** *p* < 0.001, and **** *p* < 0.0001

To determine whether persistent viral infection differentially affects glial populations, we first assessed the total number and immunological activation status of microglia, oligodendrocytes, and astrocytes one month after intracerebral MHV68 infection. While a fraction of all three cell types harbored persistent MHV68 infection (Fig. 1, A-C, Additional File 1: Fig. S1, D-I), only the microglia exhibited both a significant increase in total cell number (Fig. 2A) and robust upregulation of MHC-II expression (Fig. 2B) one month after MHV68 infection, indicating that they were activated and more immunostimulatory. This elevated MHC-II expression persisted for at least six months post-infection (Fig. 2C), demonstrating the long-term effects of persistent intracerebral infection on the microglia. In contrast, oligodendrocytes and astrocytes showed minimal changes in total cell number and MHC-II expression (Additional File 1: Fig. S4, A-C). These findings suggest that microglia are uniquely responsive to persistent viral infection and may serve as the primary antigen-presenting glial population. Based on this distinct immunological profile, we focused our subsequent analyses on microglia.

Following neurotropic and systemic infections, microglia can become “primed,” a state of heightened reactivity characterized by altered morphology, increased class II HLA expression, and an exaggerated inflammatory response upon re-stimulation [51–53]. Primed microglia have been implicated in exacerbating neuroinflammation and neurodegeneration in various neurodegenerative diseases, including MS [54–57]. The mechanisms responsible for microglial priming are largely unknown, although infections, particularly in early life, are postulated to play a role in this phenomenon [58–61]. To determine whether persistent intracerebral MHV68 infection can elicit this microglial state, we isolated whole brain cells from infected mice one month post-infection and treated them with LPS. Microglia from infected mice produced significantly higher levels of intracellular tumor necrosis factor alpha (TNF-α) upon stimulation with LPS but not at baseline, confirming that these microglia were primed (Fig. 2D). The increased TNF-α expression in the microglia of infected mice after stimulation was associated with increased absolute numbers of infiltrating immune cells, further supporting the relationship between microglial activity and CNS inflammation (Fig. 2E).

Notably, YFP-positive microglia were present within the demyelinated lesions of MOG-challenged, MHV68-infected mice (Fig. 2F), suggesting a potential role for infected microglia in lesion development. To investigate whether there are functional differences between microglia with persistent MHV68 infection and uninfected microglia, we examined YFP-positive infected and YFP-negative bystander microglia from MHV68-H2bYFP-infected mice one month post-infection. Relative to microglia in mock-infected mice, YFP-negative microglia in infected mice displayed higher levels of MHC-II protein, indicating that CNS infection affects bystander microglia (Fig. 2G). Nonetheless, YFP-positive microglia in infected mice expressed the highest level of MHC-II protein (Fig. 2G). Furthermore, YFP-positive microglia expressed substantially higher levels of MHC-II protein than YFP-positive astrocytes and oligodendrocytes, further supporting their role as the major antigen-presenting cell population in the infected CNS (Additional File 1: Fig. S4D). Upon stimulation with LPS, YFP-negative microglia from infected mice demonstrated intracellular TNF-α levels comparable to microglia from mock-infected mice, whereas TNF-α was robustly induced in YFP-positive microglia (Fig. 2H). In summary, these data demonstrate that persistent MHV68 infection of the brain primes virus-harboring microglia, potentially sensitizing the brain to subsequent inflammatory insults.

### MHV68 + BV2 cells model MHV68-induced microglial priming

**Fig. 3:**
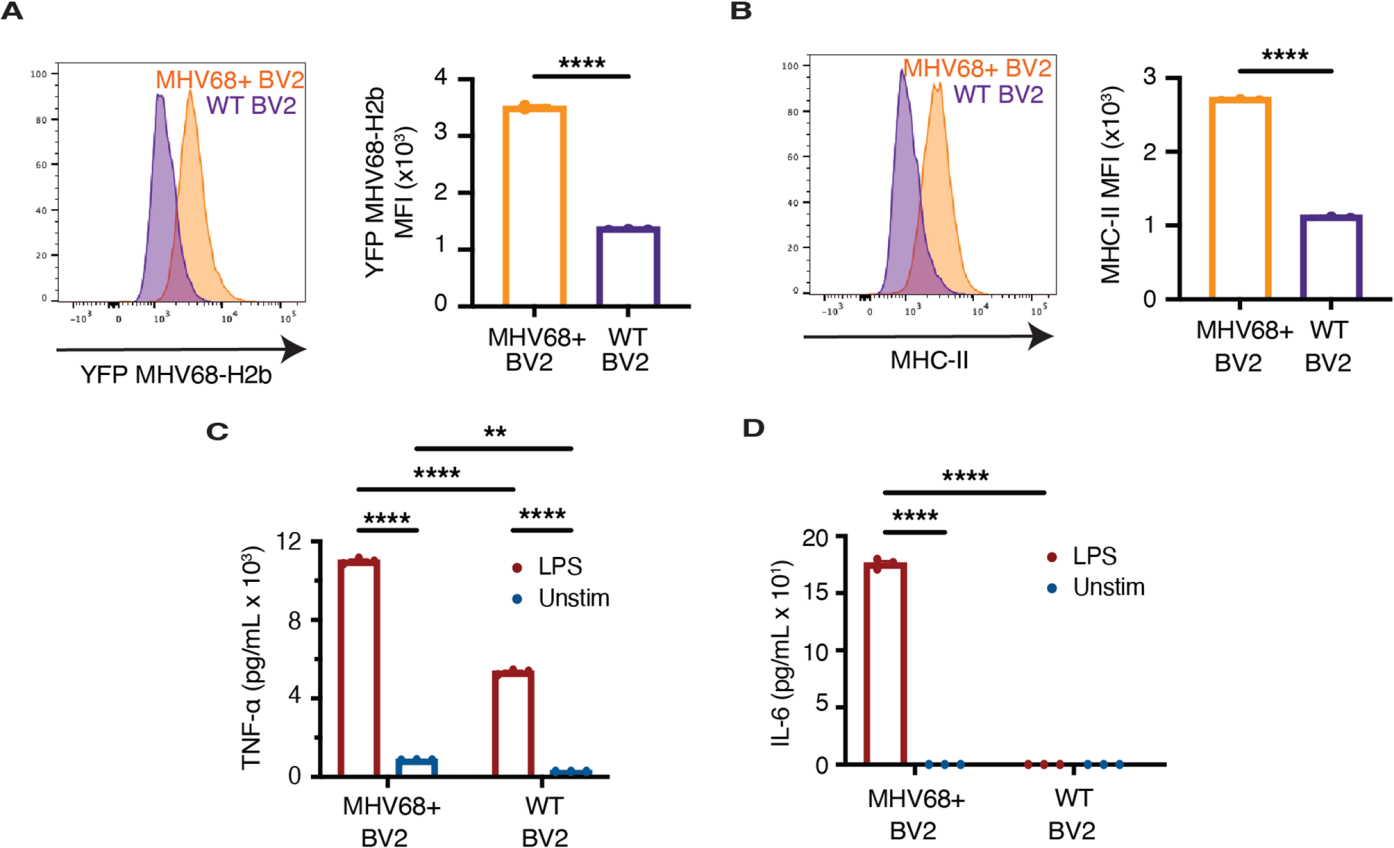
MHV68 + BV2 cells model MHV68-induced microglial priming. **A** MHV68-H2bYFP and (**B**) MHC-II expression in MHV68 + BV2 and uninfected (WT) BV2 cells (*n* = 3 technical replicates per group). Concentrations of secreted TNF-α (**C**) and IL-6 (**D**) from MHV68 + BV2 and uninfected (WT) BV2 cells (*n* = 3 technical replicates per group) following stimulation with LPS. Statistical significance was determined using an unpaired 2-tailed *t* test in **A** and **B** and a two-way ANOVA with Šidák correction for multiple comparisons in **C** and **D**. Data are represented as mean ± standard error of mean (SEM). * *p* < 0.05, ** *p* < 0.01, *** *p* < 0.001, and **** *p* < 0.0001

While intracerebral MHV68 infection results in persistent infection of microglia, similar to microglia infected with EBV in MS patients [29], the low proportion of persistently infected microglia in vivo hinders the detailed mechanistic studies of these cells. To enable such studies, we developed an in vitro model of microglia persistently infected with MHV68 by infecting the BV2 murine microglial cell line with MHV68-H2bYFP and isolating the YFP-positive (MHV68+) fraction by FACS. MHV68 + BV2 cells maintained their viability and retained their MHV68-H2bYFP expression for over 10 passages, and over several freeze-thaw cycles (Fig. 3A). We next sought to determine whether MHV68 + BV2 cells display the virus-induced microglial priming observed in vivo. Indeed, compared with uninfected cells, MHV68 + BV2 cells exhibited heightened MHC-II expression (Fig. 3B) and secreted higher levels of TNF-α (Fig. 3C) and interleukin 6 (IL-6) (Fig. 3D) upon stimulation with LPS. Thus, MHV68 + BV2 cells faithfully recapitulate the phenomenon of microglial priming observed in vivo after persistent intracerebral MHV68 infection.

### Virus-infected microglia drive increases in CD4 + T cell proliferation and pro-inflammatory cytokine production in the brain

**Fig. 4:**
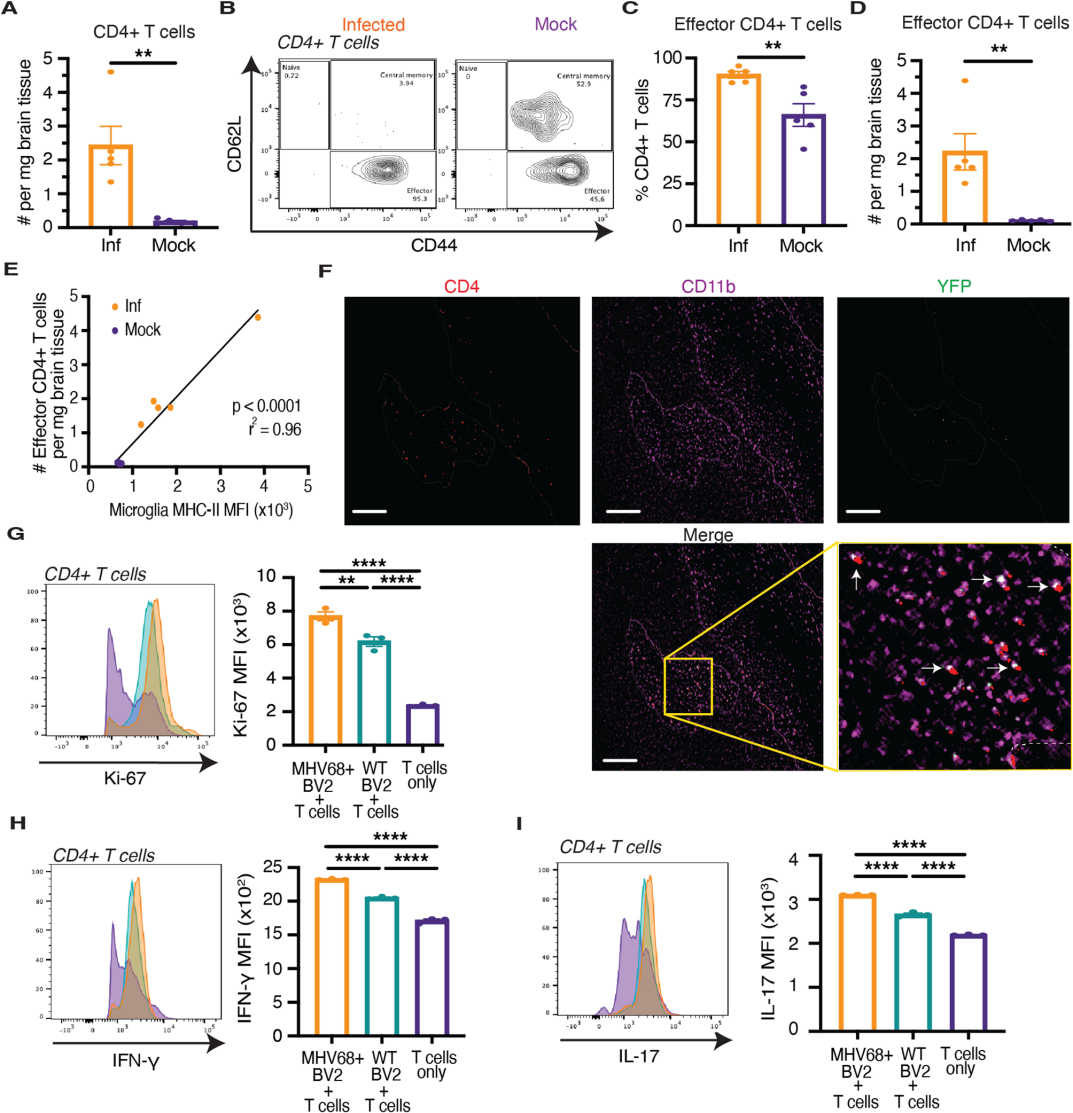
Virus-infected microglia drive increases in T cell proliferation and pro-inflammatory cytokine production in the brain. **A** Absolute number of brain-infiltrating CD4 + T cells in mice on day 30 following intracerebral MHV68 (Inf, *n* = 5) or mock infection (*n* = 5). **B** On day 30 following intracerebral MHV68 (Inf, *n* = 5) or mock infection (*n* = 5), the (**C**) frequency and (**D**) absolute number of effector CD4 + T cells isolated from the brain were determined by flow cytometry. **E** Correlation between microglial MHC-II expression and the absolute number of brain-infiltrating effector CD4 + T cells on day 30 following intracerebral MHV68 (Inf) or mock infection. **F** CD4, CD11b, and MHV68-H2bYFP staining in a demyelinated lesion from the brain of a MHV68H2bYFP-infected mouse 15 days after MOG peptide administration. Demyelinated lesion borders are marked with dashed line, and CD4 + T cells around YFP + microglia are marked with arrows. Scale bar, 50 μm. **G**-**I** MHV68 + BV2 and uninfected (WT) BV2 cells were pulsed with MOG peptide for two hours, stimulated with LPS for an additional two hours, and then co-cultured for 18 h with MOG_35−55_-primed CD4 + T cells enriched from the spleens of female C57Bl/6J mice 8 days following MOG peptide administration. Intracellular expression of (**G**) Ki-67, (**H**) IFN-γ and (**I**) IL-17 were determined by flow cytometry (*n* = 3 per group). Statistical significance was determined using an unpaired 2-tailed *t* test in **A**, **C**, and **D**, a simple linear regression in **E**, and an ordinary one-way ANOVA with Tukey’s correction for multiple comparisons in **G**-**I**. Data are represented as mean ± standard error of mean (SEM). * *p* < 0.05, ** *p* < 0.01, *** *p* < 0.001, and **** *p* < 0.0001

Active MS lesions are characterized by increased T cell infiltration and interaction of these T cells with microglia, which influence T cell activation through antigen presentation and cytokine production [62–65]. We evaluated whether similar changes occur in mice with persistent intracerebral MHV68 infection and indeed observed greater CD4 + and CD8 + T cell infiltration into the brain at one month post-infection relative to mock-infected mice (Fig. 4A, Additional File 1: Fig. S5A). Additionally, 95% of infiltrating T cells exhibited an effector phenotype, characterized by CD44 + CD62L- expression, indicative of an activated state (Fig. 4, B-D, Additional File 1: Fig. S5, B-D). Furthermore, mice with the highest microglial priming, as measured by increased microglial MHC-II expression, had the greatest number of infiltrating effector T cells (Fig. 4E, Additional File 1: Fig. S5E). CD4 + T cells within the demyelinated lesions of MOG-challenged, MHV68-infected mice were in close proximity to predominantly YFP + microglia (Fig. 4F), suggesting that persistently infected microglia may directly interact with and activate T cells.

To characterize the direct effects of primed microglia on CD4 + T cell activation, we co-cultured BV2 cells with persistent MHV68 infection—previously pulsed with MOG peptide and stimulated with LPS—with syngeneic MOG_35−55_-primed CD4 + T cells. First, a greater proportion of CD4 + T cells proliferated, as determined by intracellular Ki-67 staining, when cultured with MHV68 + BV2 cells compared to culturing with wildtype BV2 cells (Fig. 4G). In addition, MHV68 + BV2 cells induced higher intracellular interferon-gamma (IFN-γ) and IL-17 cytokine expression, which are key mediators of MS pathology [66, 67], in the CD4 + T cells compared to wildtype BV2 cells (Fig. 4, H and I). Together, these data demonstrate the enhanced ability of microglia with persistent MHV68 infection to activate CD4 + T cells and drive their differentiation towards pathogenic T helper (Th)1 and Th17 subsets.

### Persistent MHV68 infection increases the intracellular labile iron pool and alters metabolism in microglia

**Fig. 5:**
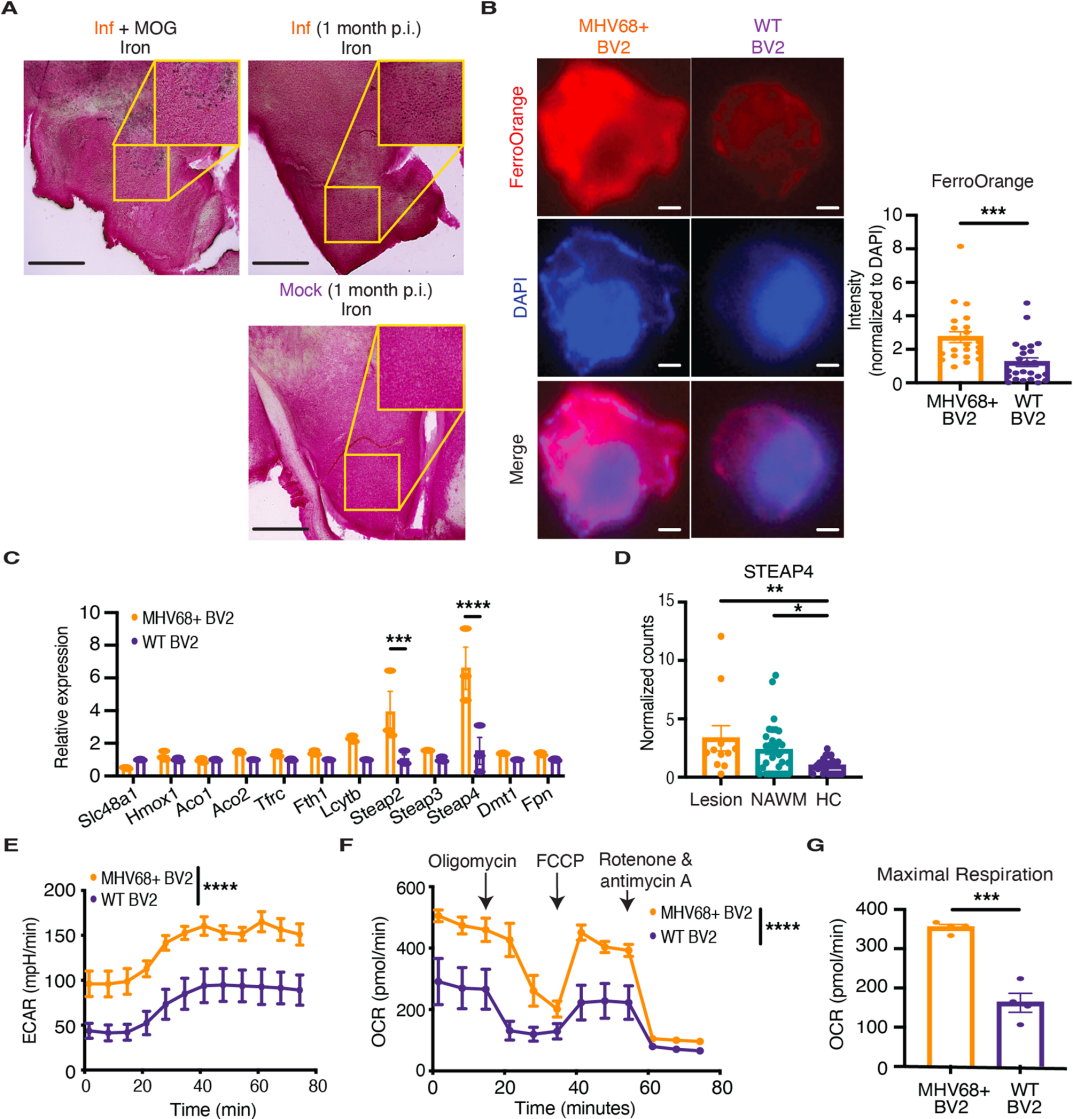
Persistent MHV68 infection increases the intracellular labile iron pool and alters metabolism in microglia. **A** Fixed frozen brain sections from MHV68-infected (Inf) or mock-infected mice one month after infection or 15 days after MOG peptide administration were stained for iron. Scale bar, 1000 μm. **B** FerroOrange staining levels in MHV68 + BV2 (*n* = 23 cells) and uninfected (WT) BV2 (*n* = 24 cells) cells using fluorescence microscopy. Scale bar, 5 μm. **C** The relative expression of genes involved in heme metabolism (*Slc48a1* and *Hmox1*), TCA cycle (*Aco1* and *Aco2*), iron transport (*Tfrc* and *Fth1*), ferrireductases (*Lcytb*, *Steap2*, *Steap3*, and *Steap4*), iron uptake (*Dmt1*), and iron export (*Fpn*) in MHV68 + BV2 and uninfected (WT) BV2 cells (*n* = 3 technical replicates per group). **D** Normalized counts of *STEAP4* in the microglia present in lesions (*n* = 11) and normal-appearing white matter (NAWM, *n* = 31) of MS patients and of healthy control (HC, *n* = 26) brain tissue. The levels of (**E**) extracellular acidification rate (ECAR) and (**F** and **G**) oxygen consumption rate (OCR) over time in MHV68 + BV2 and uninfected (WT) BV2 (*n* = 3 technical replicates per group) were determined by Agilent Seahorse XFp Mito Stress test. Statistical significance was determined using an unpaired 2-tailed *t* test in **B** and **G**, a two-way ANOVA with Šidák correction for multiple comparisons in **C**, **E**, and **F**, and an ordinary one-way ANOVA with Tukey’s correction for multiple comparisons in **D**. Data are represented as mean ± standard error of mean (SEM). * *p* < 0.05, ** *p* < 0.01, *** *p* < 0.001, and **** *p* < 0.0001

Viral infections can disrupt iron homeostasis at the tissue level, resulting in abnormal iron accumulation in the brain [68, 69]. To determine whether persistent MHV68 infection alters iron deposition in the brain, we performed histochemical staining for iron in MHV68-infected and mock-infected mice. Iron levels were increased in the brains of infected mice, indicating infection-induced iron accumulation, which became even more pronounced following MOG peptide administration (Fig. 5A). Given that iron accumulation in microglia has been observed in MS—particularly in chronic active lesions [70–72]—we next investigated whether persistently infected microglia exhibited altered iron metabolism. We measured intracellular iron (II) levels in MHV68 + BV2 and wildtype BV2 cells by FerroOrange staining. MHV68 + BV2 cells stained more strongly for FerroOrange compared to wildtype BV2 cells (Fig. 5B), suggesting that intracellular iron increases in microglia with persistent MHV68 infection. Moreover, quantitative reverse transcription polymerase chain reaction (qRT-PCR) analysis revealed higher gene expression of the ferrireductases *Steap2* and *Steap4*, which reduce iron (III) to bioavailable iron (II) [73], in MHV68 + BV2 cells relative to wildtype BV2 cells (Fig. 5C). Analysis of publicly available bulk RNA sequencing data [74] revealed that many iron metabolism genes, including *STEAP4*, were significantly upregulated in microglia residing in both the NAWM and lesions of MS patients compared to microglia in healthy control brain tissue (Fig. 5D, Additional File 1: Fig. S6), suggesting that altered iron metabolism in microglia may also play a role in MS development.

Iron is also an essential cofactor in various metabolic pathways, influencing the function and inflammatory state of microglia [75–78]. To evaluate how persistent infection affects microglial metabolism, we performed the Seahorse Mito Stress test on MHV68 + BV2 and uninfected BV2 cells. MHV68 + BV2 cells exhibited sustained elevation in glycolysis as determined by the increased extracellular acidification rate (Fig. 5E). The oxygen consumption rate was also elevated in MHV68 + BV2 cells (Fig. 5F), demonstrating that these persistently infected cells utilize OXPHOS more and have a greater spare respiratory capacity (Fig. 5G) than uninfected cells. Together, these data demonstrate that persistent viral infection drives alteration of iron metabolism in microglia, which may contribute to the metabolic changes required for the primed microglial state.

### Iron chelation dampens MHV68-induced microglial priming but aggravates MOG peptide-induced ataxia through viral reactivation

**Fig. 6:**
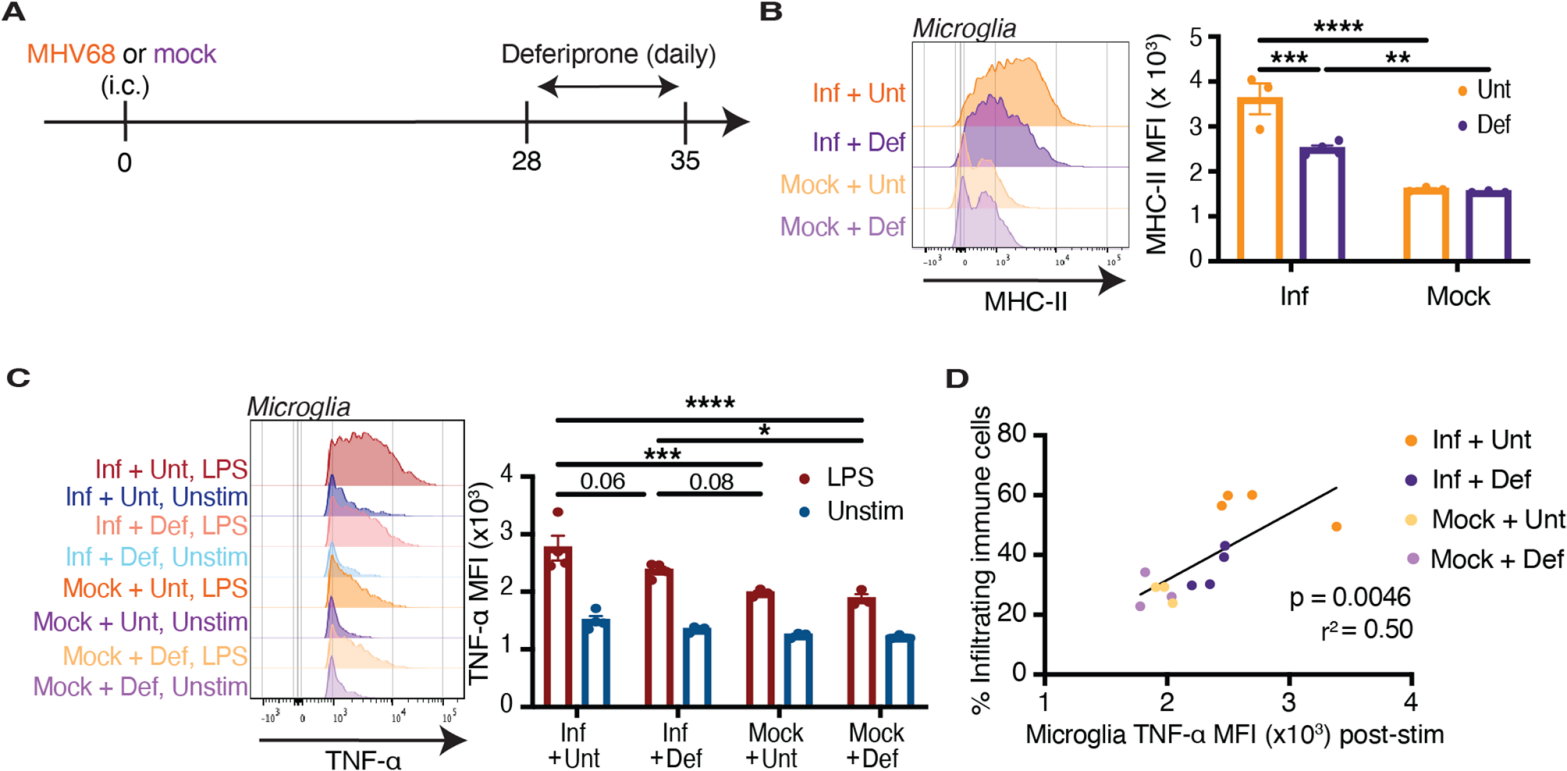
Iron chelation dampens MHV68-induced microglial priming. **A** Schematic illustration of treatment course. Mice were intracerebrally infected with MHV68 (Inf, *n* = 7) or mock-infected (*n* = 6) and pre-treated with 75 mg/kg deferiprone (Def, *n* = 7) or PBS (Unt, *n* = 6) daily for 7 days starting one month post-infection. **B** MHC-II and (**C**) TNF-α (at baseline and upon stimulation with LPS) in microglia from deferiprone-treated (Def, *n* = 7) or untreated (Unt, *n* = 7) mice on day 35 following intracerebral MHV68 (Inf, *n* = 8) or mock infection (*n* = 6). **D** Correlation between microglial TNF-α expression post-stimulation and the frequency of brain-infiltrating immune cells in deferiprone-treated or untreated mice after intracerebral MHV68 or mock infection. Statistical significance was determined using a two-way ANOVA with Šidák correction for multiple comparisons in **B** and **C**, and a simple linear regression in **D**. Data are represented as mean ± standard error of mean (SEM). * *p* < 0.05, ** *p* < 0.01, *** *p* < 0.001, and **** *p* < 0.0001

Altered iron metabolism in persistently infected microglia suggests that modulation of iron metabolism may rescue microglia from their primed state and reduce susceptibility to brain-targeted inflammation. Indeed, treatment with the intracellular iron chelator, deferiprone [79], downregulated *Steap4* expression in MHV68 + BV2 cells (Additional File 1: Fig. S7A). Deferiprone increased MHC-II expression (Additional File 1: Fig. S7B) but decreased the secretion of TNF-α (Additional File 1: Fig. S7C) and IL-6 (Additional File 1: Fig. S7D) in MHV68 + BV2 cells. To evaluate the effect of iron chelation in vivo, we treated MHV68-infected and mock-infected mice with deferiprone daily starting one month post-infection (Fig. 6A). After seven consecutive days, priming had decreased in the microglia of infected mice, based on diminished MHC-II expression (Fig. 6B) and a reduction in intracellular TNF-α following stimulation (Fig. 6C), suggesting that modulation of iron metabolism can partially rescue microglia from their MHV68-induced primed state. The decrease in TNF-α expression in the microglia of deferiprone-treated MHV68-infected mice after stimulation was associated with a decrease in the frequency of infiltrating immune cells (Fig. 6D).

To assess the efficacy of deferiprone in dampening MOG peptide-induced ataxia, one month post-infection, we treated mice with deferiprone or PBS for seven consecutive days, and then administered MOG peptide to induce EAE, continuing treatment through EAE development. Deferiprone treatment aggravated ataxia in infected mice (Additional File 1: Fig. S8A). Intracellular iron chelation has been demonstrated to reactivate the EBV lytic cycle [80], which can induce cerebellar ataxia [81]. To determine whether deferiprone reactivates MHV68 in microglia, we treated MHV68 + BV2 cells with deferiprone and found that deferiprone upregulated MHV68-H2bYFP expression (Additional File 1: Fig. S8B) and increased MHV68 secretion (Additional File 1: Fig. S8C), indicating viral reactivation. Together, these data show that while iron chelation may rescue microglia from their primed state, it aggravates brain-targeted inflammation through viral reactivation.

### Early antiviral treatment following intracerebral MHV68 infection reduces microglial priming and prevents MOG peptide-induced ataxia

**Fig. 7:**
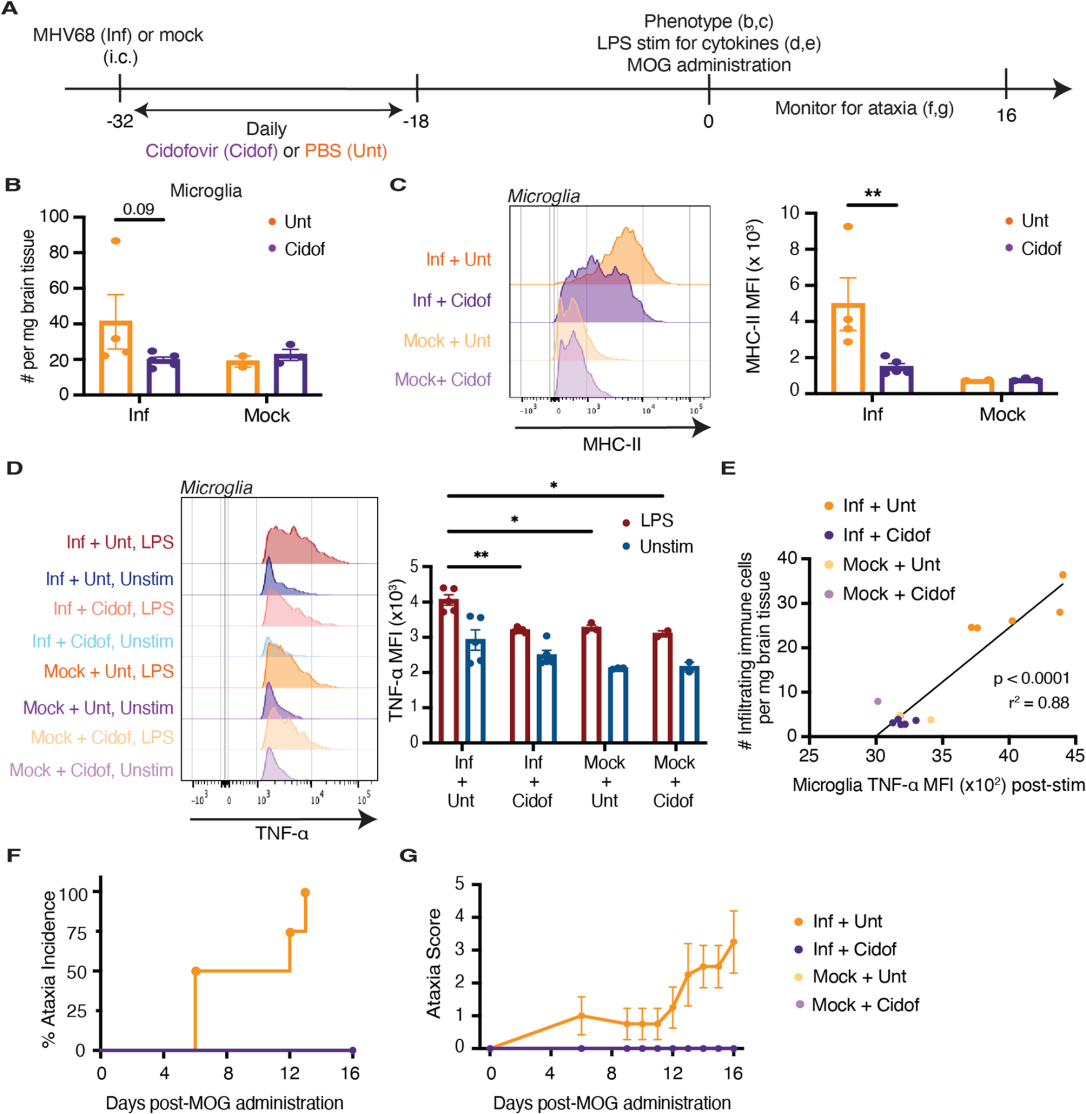
Early antiviral treatment following intracerebral MHV68 infection reduces microglial priming and prevents MOG peptide-induced ataxia. **A** Schematic illustration of antiviral treatment course starting two hours after intracerebral MHV68 or mock infection. **B** Absolute number of microglia on day 30 following intracerebral MHV68 (Inf, *n* = 9) or mock infection (*n* = 5) and daily treatment with cidofovir (Cidof, *n* = 8) or PBS (Unt, *n* = 6) starting two hours after infection. **C** MHC-II expression on microglia in cidofovir-treated (Cidof, *n* = 8) or untreated (Unt, *n* = 6) mice on day 30 after intracerebral MHV68 (Inf, *n* = 9) or mock infection (*n* = 5). **D** Intracellular TNF-α expression, at baseline and upon stimulation with LPS, in microglia isolated from cidofovir-treated (Cidof, *n* = 7) or untreated (Unt, *n* = 8) mice on day 30 after intracerebral MHV68 (Inf, *n* = 10) or mock infection (*n* = 5). **E** Correlation between microglial TNF-α expression post-stimulation and the absolute number of brain-infiltrating immune cells from cidofovir-treated and untreated mice on day 30 after intracerebral MHV68 (Inf) or mock infection. MOG peptide was administered one month following intracerebral MHV68 infection. The incidence (**F**) and severity (**G**) of ataxia following MOG peptide administration in cidofovir-treated (Cidof, *n* = 7) or untreated (Unt, *n* = 6) mice after intracerebral MHV68 (Inf, *n* = 8) or mock (*n* = 5) infection. Statistical significance was determined using a two-way ANOVA with Šidák correction for multiple comparisons in **B**, **C**, and **D**, and a simple linear regression in **E**. Data are represented as mean ± standard error of mean (SEM). * *p* < 0.05, ** *p* < 0.01, *** *p* < 0.001, and **** *p* < 0.0001

While antiviral agents can inhibit EBV replication in vitro, they have shown limited efficacy in treating the acute manifestations of EBV infection or shortening its course in the clinic [82–84]. Still, we considered the possibility that antiviral treatment may mitigate the long-term effects of infection on the brain. To evaluate this possibility, we intracerebrally infected mice with MHV68 and immediately started daily treatment with cidofovir, an acyclic nucleoside phosphonate analog shown to protect mice from lethal MHV68 infection [85], for 14 days (Fig. 7A). We isolated the brains of treated and untreated mice one month post-infection and characterized the phenotypic changes in microglia by flow cytometry. Treatment with cidofovir following intracerebral MHV68 infection did not significantly affect the number of microglia compared to untreated mice (Fig. 7B). However, cidofovir treatment reduced MHC-II expression in infected mice (Fig. 7C) and decreased TNF-α expression (Fig. 7D) to levels similar to those observed in microglia of mock-infected mice after stimulation. Additionally, the decrease in microglial TNF-α expression in treated mice after stimulation was associated with a decrease in the absolute number of infiltrating immune cells (Fig. 7E). To assess the efficacy of early cidofovir treatment in dampening MOG peptide-induced ataxia, we administered MOG peptide to mice one month following intracerebral MHV68 infection. Remarkably, infected mice treated with cidofovir did not exhibit ataxia (Fig. 7, F and G). Thus, early antiviral treatment at the time of acute intracerebral MHV68 infection diminishes virus-induced microglial priming and prevents subsequent brain-targeted inflammation following challenge with a second inflammatory stimulus.

## Discussion

MS is well-recognized to be influenced by both genetic and environmental factors, with EBV infection likely playing a key role [5]. Epidemiological evidence suggests that while EBV infection is necessary for MS development, it is not sufficient on its own, since most individuals infected with EBV do not develop MS, and those who are not infected have virtually no risk of the disease [6]. Studies using viral models of MS have shown that the route of infection can affect the extent of resulting tissue damage, with CNS lesions arising only following intracerebral or intranasal, but not systemic, administration [31–34, 86, 87]. Despite extensive research on the impact of systemic EBV infection, to our knowledge no studies have examined whether intracerebral EBV infection can induce chronic CNS inflammation and demyelination. In our study of the EBV-like MHV68 virus, we found that intracerebral infection alone was insufficient to induce sustained disease manifestations. Unlike TMEV, which persists only in susceptible mouse strains (e.g., SJL/J) but not in resistant ones (e.g., C57BL/6) [31], MHV68 was able to persist in the brains of C57BL/6 mice. Furthermore, mice with persistent intracerebral MHV68 infection developed brain-targeted inflammation and demyelination following the induction of myelin-specific T cells by MOG peptide. These findings suggest that intracerebral MHV68 may prime brain-targeted inflammation differently than viruses unrelated to EBV that typically induce only spinal cord-targeted inflammation. Thus, our MHV68 model may be a more informative model in which to study the mechanisms driving EBV’s role in MS development.

B cells are considered the primary latent reservoir of EBV [88], but EBV also persists in other immune cells, including macrophages in EBV-related tumors [20] and chronic active EBV infection [21], and microglia, the resident macrophages of the CNS, in MS [29, 30]. In our study, we found that MHV68 also persistently infected microglia in vivo, consistent with studies showing MHV68 infects primary microglia and microglial cell lines in vitro [89, 90]. Moreover, this persistent infection immunologically primed the microglia, leading to upregulation of MHC-II expression and exaggerated secretion of TNF-α upon re-stimulation. Persistently infected microglia were localized within demyelinated lesions and closely interacted with T cells, suggesting a potential role in directly promoting demyelination and associated motor deficits. These findings parallel observations in human immunodeficiency virus type 1 (HIV-1) infection, where the virus persists in microglia and directly enhances IL-1β expression via viral protein Tat-driven activation of the NLRP3 inflammasome, contributing to HIV-1-associated neurocognitive disorders [91, 92]. Microglial priming is a well-recognized feature of various neurodegenerative diseases [51–54], and our results suggest that persistent CNS infection with an EBV-like virus drives similar changes in microglia—potentially in a manner more potent and longer-lasting than systemic infection.

Alterations in iron metabolism within the CNS are commonly observed in MS [93–95], leading to iron accumulation particularly in microglia residing in paramagnetic rim lesions, a subset of chronic active MS lesions [70–72]. However, the precise mechanism driving iron accumulation in microglia and the impact of such accumulation on MS progression remain unclear. In our study, we found that persistent MHV68 infection upregulated *Steap4*, a ferrireductase that contributes to Th17-mediated neuroinflammation [96], in microglia, consistent with upregulation of ferrireductases in B cells latently infected with EBV [97]. This suggests a potential link between iron accumulation, chronic microglial priming, and brain-targeted inflammation, aligning with the observed correlation between iron buildup in lesions and disease progression in MS [98, 99]. However, while iron chelation reduced *Steap4* expression and alleviated microglial priming, it also reactivated the viral replication cycle in microglia. This suggests that iron accumulation may also represent a protective mechanism to limit viral spread in the CNS, which is consistent with the notion that innate immune cells sequester iron to reduce circulating iron levels and restrict viral dissemination [73]. Thus, the role of iron appears context-dependent: it may be protective in limiting virus-induced inflammation, but detrimental in promoting chronic neuroinflammation under certain conditions, such as in the presence of autoreactive T cells after MOG peptide administration. This intricate balance may help explain the mixed results observed in clinical trials of deferiprone as a treatment for MS [100]. Notably, although we initially hypothesized that iron chelation would reduce MOG peptide-induced neuroinflammation by reversing microglial priming, treatment unexpectedly worsened disease severity, underscoring the complexity of targeting iron metabolism in the context of prior viral infection.

The effects of iron chelation on viral reactivation underscore the need for a deeper understanding of viral gene expression dynamics under different conditions. While studies of MHV68 gene expression in myeloid cells are limited, one study showed that the viral gene transcription program in macrophages differs from the latent programming in infected B cells and the lytic gene expression in infected epithelial cells [101]. This suggests that the interaction between latency and lytic replication in microglia may be more complex than in other cell types, which is why we classified cells as persistently infected instead of latently or lytically infected. In the context of EBV, latent gene expression has been detected in the white matter and meninges, whereas lytic gene expression is primarily localized to chronic MS lesions [27]. This may point to a role for EBV latency in the early stages of MS development, while lytic replication becomes more prominent in the later stages of lesion formation. Further research is needed to determine how different viral programming states affect the brain, particularly the microglia.

The long-term effects of MHV68 infection on microglial function and neuroinflammation highlight the need for improved therapeutics targeting persistent EBV infection and its sustained effects on microglia. Iron chelation, an already proposed lytic induction therapy to reactivate latent virus and increase susceptibility to immune-mediated viral clearance [102–104], has shown some promise, but our study reveals that it can also induce virus-related pathology. More studies will be needed to evaluate whether other therapies aimed at repolarizing microglia and macrophages have similar effects. While no current antivirals can prevent latent EBV infection in the clinic [82–84], our findings demonstrate that early antiviral treatment may be the most effective strategy to mitigate the development of virus-induced microglial priming and neuropathology, suggesting that similar effects could be achieved for EBV [105]. Vaccination remains a promising strategy to prevent primary infection, although EBV’s ability to establish latency complicates this approach [106–109]. Small molecule inhibitors targeting viral replication and latency maintenance show promise in preclinical studies [110–113]. Adoptive T cell therapy targeting EBV may offer a way to target and eliminate EBV-infected cells [114, 115], but its potential to treat or reverse MS remains unclear. Together, strategies to prevent primary EBV infection or eliminate latent viral reservoirs may be the most effective at preventing or dampening MS development and progression.

We found that MHV68 also infected brain-resident glial cells, including astrocytes and oligodendrocytes, aligning with recent reports that EBV-infected glial cells are present in MS lesions [30]. To our knowledge, the effects of persistent EBV infection on these cell types have not been systemically studied. Astrocytes and oligodendrocytes play crucial but distinct roles in MS pathogenesis. Astrocytes contribute to neuroinflammation by producing pro-inflammatory cytokines and chemokines that recruit and activate immune cells, and by forming glial scars that inhibit remyelination [116]. Although not classical antigen-presenting cells, astrocytes can also express MHC class II and co-stimulatory molecules under inflammatory conditions, though their antigen-presenting capacity is more limited and context-dependent than that of microglia [117]. In our model, astrocytes did not exhibit persistent MHC-II expression, but they may contribute to demyelination through mechanisms not related to antigen presentation. Oligodendrocytes, the myelin-producing cells of the CNS, are directly targeted by the immune system in MS, directly leading to demyelination [118]. In progressive MS, failure of oligodendrocyte precursor cells to differentiate and remyelinate axons further exacerbates disease progression [118]. Notably, neurotropic mouse hepatitis virus can persistently infect oligodendrocytes in mice and disrupt their ability to remyelinate [119]. These findings underscore the need to further investigate how persistent EBV or MHV68 infection affects individual CNS cell types, which may reveal new therapeutic targets for MS.

The etiology of many neurological diseases, including MS, remains unclear, but viral infections have been associated with an increased risk of developing these conditions [120]. In particular, immune changes induced by persistent EBV infection have been implicated in the development of not only MS, but also Alzheimer’s and Parkinson’s disease [121, 122]. EBV reactivation may exacerbate neurological symptoms in patients with long COVID [123]. Additionally, there are conflicting reports whether EBV may also trigger myelin oligodendrocyte glycoprotein-associated disease (MOGAD), a distinct inflammatory demyelinating disease driven by MOG-specific antibodies [124, 125]. While our model was developed in the context of MS, it may also have relevance to MOGAD, as virus-primed microglia may exhibit enhanced sensitivity to antibody-mediated inflammation within the CNS. Our findings suggest that virus-induced microglial priming, mediated by alterations in iron metabolism, could represent a shared mechanism underlying these different conditions. Notably, we observed that lesions were more prominent in a similar region of the brain where mice were initially infected, suggesting that the long-term consequences of viral infection may vary depending on the affected brain region. For instance, while hindbrain involvement may predispose to demyelinating or motor disorders like our model, persistent infection in the hippocampus or cerebral cortex could plausibly contribute to Alzheimer’s-like pathology. Thus, persistent priming of microglia following brain infection may sensitize certain CNS regions to subsequent insults, that need not be infectious in nature but can result in widespread neuroinflammation and disease. If so, further investigation into virus-induced microglial dysfunction may uncover new therapeutic targets for MS and other CNS-related diseases, ultimately improving patient outcomes.

## Conclusions

In summary, our findings suggest that persistent gammaherpesvirus infection in microglia may play an important role in brain dysfunction and increase susceptibility to neuroinflammatory diseases. The virus persists in microglia, inducing a primed microglial state implicated in many neurodegenerative diseases while simultaneously creating a viral reservoir that can trigger neuropathology upon reactivation. Unlike other viruses used in common mouse models of MS, gammaherpesvirus infection in the CNS requires additional inflammatory stimuli to induce chronic neurological effects, supporting a potential two-step mechanism by which EBV infection of the CNS can lead to MS. These findings provide a compelling rationale for further investigation of this two-step mechanism, with the goal of developing targeted therapeutics to intervene at either step and prevent MS development. However, future therapeutic approaches will likely need to carefully balance modulation of infected cells and reactivation of the virus. Our novel viral mouse model of MS presents a valuable tool for advancing the understanding of this two-step mechanism and for evaluating potential strategies to prevent or mitigate the development of MS, as well as other neurodegenerative diseases.

## Methods

### Mice

Female 5–6 week old C57Bl/6J mice (JAX #000664) were purchased from the Jackson Laboratory. Mice were housed in a designated ABSL2 animal facility at Stanford University on a 12-hour light/dark cycle.

### Virus generation and administration

MHV68 (murid herpesvirus 4, ATCC, VR-1465) was obtained from ATCC and MHV68-H2bYFP [48] was provided by Dr. Laurie Krug, and stocks were prepared by infection of BHK-21 cells (hamster kidney cells, ATCC, CCL-10) as described previously [126]. Viral plaque assays were performed on BHK-21 cells using carboxymethyl cellulose overlays to determine titer of MHV68 stocks as described previously [127]. Intracerebral infection was performed using a stereotaxic frame. Prior to infection, 7–8 week old mice were anesthetized with ketamine/xylazine and given buprenorphine ER as analgesic. Using a small drill, a hole was made in the skull and 10 plaque forming units of virus was injected into the midbrain in 2.5 µL of serum-free Dulbecco’s Modified Eagle Medium (DMEM) using a Hamilton syringe. After injection, the syringe was withdrawn slowly, the hole in the skull was covered with bone wax (Braintree Scientific), and then the skin was sutured. Mice were checked daily after surgery.

### Demyelinating disease scoring

Demyelinating disease symptoms were scored as follows: 0, normal; 1, slight waddling gait; 2, waddling gait; 3, spastic hind limb paralysis; 4, severe hindlimb paralysis. These scores have been described previously [128] and shown to be indicative of demyelination for the TMEV-IDD model.

### Experimental autoimmune encephalomyelitis (EAE) induction

EAE was induced in female 11–12 week old C57Bl/6J mice, about one month post-infection, using the Hooke Kit MOG_35−55_/CFA Emulsion PTX (Hooke Laboratories, EK-2110). Mice were anesthetized with ketamine/xylazine and then injected subcutaneously with 100 µL of MOG_35−55_/CFA Emulsion in two sites, the upper and lower back (total of 200 µL). Four hours later, mice were intraperitoneally injected with 200 ng of pertussis toxin (PTX) in 100 µL of freshly prepared solution in Phosphate Buffered Saline (PBS). PTX solution was administered again 24 h later. When mice developed more severe EAE (classical EAE score ≥ 3.0), we added DietGel Boost (CleanH_2_O) and administered subcutaneous fluids daily.

### Classical EAE scoring

Classical EAE symptoms were scored as follows: 0, normal; 0.5, limp tip of tail; 1.0, limp tail; 1.5, hindlimb inhibition; 2.0, weakness of hindlimbs; 2.5, dragging of hindlimbs; 3.0, complete paralysis of hindlimbs; 3.5, complete hindlimb paralysis and inability to right itself; 4.0, complete hindlimb paralysis and partial forelimb paralysis; 4.5, complete hindlimb and partial forelimb paralysis and no movement around cage; 5.0, moribund. After euthanasia, mice were given a score of 5.0 for the remainder of the experiment.

### Ataxia scoring

Ataxia was scored using an established composite scoring system for mouse models of cerebellar ataxia. In the absence of classical EAE symptoms, scores of 0–3 were assigned for four different neurological tests: ledge test, hindlimb clasping, gait, and kyphosis. For the ledge test, mice were placed on the cage ledge and observed as they walked and descended. A score of 0 indicated normal balance and descent, while a score of 3 was assigned if the mouse fell or refused to walk along the ledge. For hindlimb clasping, we assessed the mouse’s hindlimb posture during tail suspension. A score of 0 indicated normal splayed hindlimbs, while a score of 3 indicated full retraction of both limbs. For gait, we evaluated the mouse’s walking pattern and weight support. A score of 0 indicated normal gait, while a score of 3 indicated severe impairment, such as abdominal dragging or tremor. For kyphosis, we assessed postural abnormalities due to neurodegeneration. A score of 0 indicated a straight spine during movement, while a score of 3 reflected pronounced, uncorrected spinal curvature. Additional details on the scoring rubric, including the intermediary scores, are provided in accompanying method article [129]. The sum of the scores (0–12) from the four tests was calculated for the final ataxia score

### Histology and immunohistochemistry

Mice were euthanized with CO_2_, and perfused with 20 mL PBS and 10 mL 4% paraformaldehyde (PFA, Electron Microscopy Sciences) each. The brains and spinal cords were then isolated, fixed in 4% PFA for 24 h, and cryopreserved in 30% sucrose for 4–5 days. Next, the tissues were frozen in OCT (Fisher Scientific) and 14-micron thick sections were processed and stored at -20 °C until the day of staining. For immunohistochemistry, sections were incubated at room temperature for 1 h and then washed in 1X Tris-buffered saline (TBS, pH 7.6, Fisher Scientific) for 5 min. Sections were permeabilized in 0.3% Triton-X100 (Sigma-Aldrich) for 15 min at room temperature, and then washed in TBS three times. Then, blocking solution with 1% bovine serum albumin (BSA, Sigma-Aldrich), 5% normal goat serum (ThermoFisher), and 5% normal donkey serum (Abcam) in 1X TBS with 0.05% Tween-20 (TBST, Sigma-Aldrich) was added to sections for 1 h. After three washes in TBST, sections were incubated with either 1:100 rat anti-mouse CD11b (clone M1/70, BioLegend) or rabbit anti-mouse CD4 (clone RM1013, Abcam) in blocking solution overnight at 4 °C. After 18 h, sections were washed in TBST and incubated with either 1:300 FluoroMyelin Red (Invitrogen), 1:500 goat anti-rat IgG-Cy5 (Abcam), or 1:500 donkey anti-rabbit IgG-TRITC (Abcam) for 1 h at room temperature. After three washes, sections were mounted with Vectashield Plus Antifade Mounting Medium with DAPI (Vector Laboratories). For histology, we used the Iron Stain kit (Abcam). Sections were incubated at room temperature for 1 h and then hydrated in distilled water. Sections were then incubated with a 1:1 ratio of Potassium Ferrocyanide and Hydrochloric Acid solutions (Abcam) for 3 min. After washing in distilled water, sections were stained in Nuclear Fast Red solution (Abcam) for 5 min. Sections were then rinsed four times in distilled water and mounted with Vectashield Plus Antifade Mounting Medium (Vector Laboratories). Images were then acquired using a Keyence BZ-X1000 microscope (4X objective lens).

### Focal demyelination model

One month after intracerebral infection with MHV68, mice were anesthetized with ketamine/xylazine and given buprenorphine ER as analgesic. Using a small drill, a hole was made at the same location in the skull as the injection of the virus and 10 µg of lipopolysaccharide (LPS, Invitrogen) was injected in 5 µL of PBS using a Hamilton syringe. After injection, the syringe was withdrawn slowly, the hole in the skull was covered with bone wax (Braintree Scientific), and then the skin was sutured. Brains were then isolated 7 days after LPS injection, and 14-micron thick fixed frozen sections were treated similarly to as described in the “Histology and immunohistochemistry” section, without the primary antibody staining. In brief, sections were permeabilized in 0.3% Triton-X100 for 15 min at room temperature, blocked with 1% BSA, 5% normal goat serum, and 5% normal donkey serum for 1 h at room temperature, incubated with 1:300 FluoroMyelin Red for 1 h at room temperature, and mounted with Vectashield Plus Antifade Mounting Medium with DAPI (Vector Laboratories). Images were then acquired using a Keyence BZ-X1000 microscope (4X objective lens).

### In vivo treatments

For cidofovir treatment, mice were administered 25 mg/kg cidofovir intraperitoneally for 14 consecutive days, starting a few hours after infection. EAE was induced one month post-infection. For deferiprone treatment, mice were injected with 75 mg/kg deferiprone intraperitoneally for 7 consecutive days, starting one month post-infection. After EAE induction, daily deferiprone treatment was continued for the remainder of the experiment.

### Characterization of CNS immune cells

Mice were euthanized with CO_2_, and perfused with 20 mL PBS each. The brains and spinal cords were then isolated and homogenized in complete DMEM (cDMEM) with 10% fetal bovine serum, 1% glutamine, and 1% penicillin/streptomycin using a pre-chilled Dounce homogenizer. The cell suspension was transferred to a 50 mL conical tube with a 70 μm filter and centrifuged. The pellet was resuspended in 30% Percoll in cDMEM once for the spinal cords and twice for the brains. After centrifugation, cells were labeled with Live/Dead™ Fixable Blue Dead Cell stain (Invitrogen) and Fc block (BioXcell, clone 2.4G2). The cells were then labeled with monoclonal antibodies against cell-specific surface markers, provided in Additional File 1: Table S1. For intracellular staining of TNF-α, cells were activated in vitro with 500 ng/mL LPS (Invitrogen) in the presence of 5 µg/mL brefeldin A (BioLegend) for 4 h, permeabilized in Cytofix/Cytoperm (BD Biosciences), and then stained with anti-TNF-α monoclonal antibodies. Stained cells were fixed with 1% PFA (Electron Microscopy Sciences), and then analyzed using a Fortessa Flow Cytometer. 123 count eBeads™ Counting Beads (Invitrogen) were used to calculate absolute counts. Data were analyzed using FlowJo software. Gating strategy for the different panels can be found in Additional File 1: Fig. S9-11.

### Cell lines

BV2 cells (murine microglia) were provided by Dr. Khoa Nguyen. In order to generate a persistently infected microglial cell line, we infected BV2 cells with MHV68-H2bYFP. After two weeks, the persistently infected (MHV68+) BV2 cells were sorted based on their YFP expression using fluorescence-activated cell sorting (FACS).

### Microglia-T cell co-culture

MHV68 + BV2 and wildtype BV2 cells were plated at 5 × 10^4^ cells per well in a 96-well plate in cDMEM. The BV2 cells were pulsed with 50 µg/mL of Mog_35−55_ peptide (R&D Systems) for 2 h at 37 °C, and then stimulated with 100 ng/mL LPS (Invitrogen) for 2 h at 37 °C. 2 × 10^5^ CD4 + T cells, enriched from the spleens of female C57Bl/6J mice 8 days after EAE induction using the CD4 T cell Isolation kit (Miltenyi Biotec), were co-cultured with the BV2 cells overnight at 37 °C in cDMEM. Brefeldin A (5 µg/mL, BioLegend) was added to each well for the last 2 h. After the co-culture, cells were labeled with Live/Dead™ Fixable Blue Dead Cell stain (Invitrogen) and Fc block (BioXcell, clone 2.4G2), and then labeled with monoclonal antibodies against cell-specific surface markers, provided in Supplemental Table 1. The cells were then permeabilized with Cytofix/Cytoperm (BD Biosciences), stained with monoclonal antibodies against Ki-67, IFNγ, and IL-17 A, fixed with 1% PFA (Electron Microscopy Sciences), and then analyzed using a Fortessa Flow Cytometer. Data were analyzed using FlowJo software. The gating strategy can be found in Additional File 1: Fig. S12.

### Intracellular iron staining

 MHV68 + BV2 and wildtype BV2 cells were plated at 2 × 10^5^ cells per well in a 6-well plate in cDMEM and incubated overnight at 37°C. After 16 hours, cells were stained with 1 µM BioTracker FerroOrange Live Cell Dye (Cell Signaling Technology) in serum-free DMEM for 30 minutes at 37°C. Cells were then washed, counterstained with 1 µg/mL 4’,6-diamidino-2-phenylindole (DAPI, Invitrogen), and fixed with 1% PFA (Electron Microscopy Sciences). Cells were imaged on a Keyence BZ-X810 microscope and data were analyzed using Fiji ImageJ software.

### Reverse transcription polymerase chain reaction

Total RNA was extracted from MHV68 + BV2 and wildtype BV2 cells when they reached 80–90% confluency using the RNeasy Mini Kit (Qiagen). After a DNase I reaction, reverse transcription was performed using the High-Capacity cDNA Reverse Transcription Kit (Applied Biosystems). The thermal cycling conditions consisted of 10 min at 25 °C, 120 min at 37 °C, and 5 min at 85 °C. cDNA samples were diluted in sterile DNase- and RNase-free water, and used for real-time quantitative PCR utilizing the QuantStudio Real-Time PCR system. The thermal cycling conditions included a hold stage with 2 min at 50 °C and 10 min at 95 °C, and a PCR stage with 40 cycles of 15 s at 95 °C and 1 min at 60 °C. Double delta Ct analysis was then performed, using *Gapdh* as the housekeeping gene. Primer sequences can be found in Additional File 1: Table S2.

### In vitro treatments

For deferiprone treatment, MHV68 + BV2 and wildtype BV2 cells were pre-treated with 500 µM deferiprone for 2 h, and then analyzed for gene expression, phenotype, and cytokine secretion.

### Cytokine bead array

MHV68 + BV2 and wildtype BV2 cells were plated at 2 × 10^4^ cells per well in a 96-well plate in cDMEM. BV2 cells were stimulated with 500 ng/mL LPS (Invitrogen) overnight. After 16 h, supernatant was collected and incubated with anti-TNF (BD Biosciences) and anti-IL-6 (BD Biosciences) capture and detection antibodies. Samples were then analyzed using a Fortessa Flow Cytometer. Data were analyzed using FlowJo software.

### Metabolic assays

MHV68 + BV2 and wildtype BV2 cells were plated at 5 × 10^4^ cells per well in cDMEM in a Seahorse XFp Cell Culture Miniplate. The plate was incubated overnight at 37 °C to allow the cells to adhere. The following day, growth media was exchanged with Seahorse Phenol Red-free DMEM. The XFp Mito Stress test was performed on the cells according to the manufacturer’s instructions.

### Bulk RNA sequencing analysis

The count matrix was downloaded from GSE179427 [74]. We used the R package DESeq2 on the count matrix for differential gene expression analysis. The normalized count matrix was then used to compare the expression of various iron metabolism genes in NAWM and lesions of MS patients and brain tissue from healthy controls. For gene set enrichment analysis, we applied the normalized count matrix to the R package “fgsea”, specifically analyzing the hallmark pathway “GOBP: Intracellular Iron Ion Homeostasis”.

### Statistical analyses

All statistical analyses were carried out using GraphPad Prism 10 software. All analyses with 2 groups were tested using unpaired 2-tailed *t* tests. All analyses with 3 groups were tested with an ordinary one-way ANOVA with Tukey’s correction for multiple comparisons. All analyses with two independent variables were tested with a two-way ANOVA with Šidák multiple comparisons. All analyses with disease incidence were tested with a log-rank test. All analyses with correlations were tested using simple linear regressions. A *p* value less than 0.05 (*) was considered significant; ** *p* < 0.01, *** *p* < 0.001, and **** *p* < 0.0001.

## Supplementary Information

Additional file 1: Supplementary Figures (Fig. S1-12) and Tables (Table S1-2).

## Data Availability

No datasets were generated or analysed during the current study.

## Abbreviations

MS: Multiple sclerosis
EBV: Epstein-Barr virus
CNS: Central nervous system
TMEV: Theiler’s murine encephalomyelitis virus
MHV: Murine hepatitis virus
SFV: Semliki forest virus
EAE: Experimental autoimmune encephalomyelitis
NAWM: Normal-appearing white matter
MHV68: Murine gammaherpesvirus-68
YFP: Yellow fluorescent protein
MOG: Myelin oligodendrocyte glycoprotein
TMEV-IDD: Theiler’s murine encephalomyelitis virus-induced demyelinating disease
HLA: Human leukocyte antigen
MHC-II: Major histocompatibility complex class II
LPS: Lipopolysaccharide
TNF-α: Tumor necrosis factor alpha
TCR: T cell receptor
IL: Interleukin
IFN-γ: Interferon-gamma
Th: T helper
qRT-PCR: Quantitative reverse transcription polymerase chain reaction
OXPHOS: Oxidative phosphorylation
HIV-1: Human immunodeficiency virus type 1
MOGAD: Myelin oligodendrocyte glycoprotein-associated disease
