# Supplementary material for: Early antiviral treatment following gammaherpesvirus-68 infection of the central nervous system prevents subsequent multiple sclerosis-like disease
Source: J Neuroinflammation. 2025 Oct 8;22:228. doi: 10.1186/s12974-025-03547-8 (PMC12509357; doi:10.1186/s12974-025-03547-8)
Supplement: Supplementary file 1 — Additional file 1: Supplementary Figures (Fig. S1-12) and Tables (Table S1-2). [file 12974_2025_3547_MOESM1_ESM.pdf]

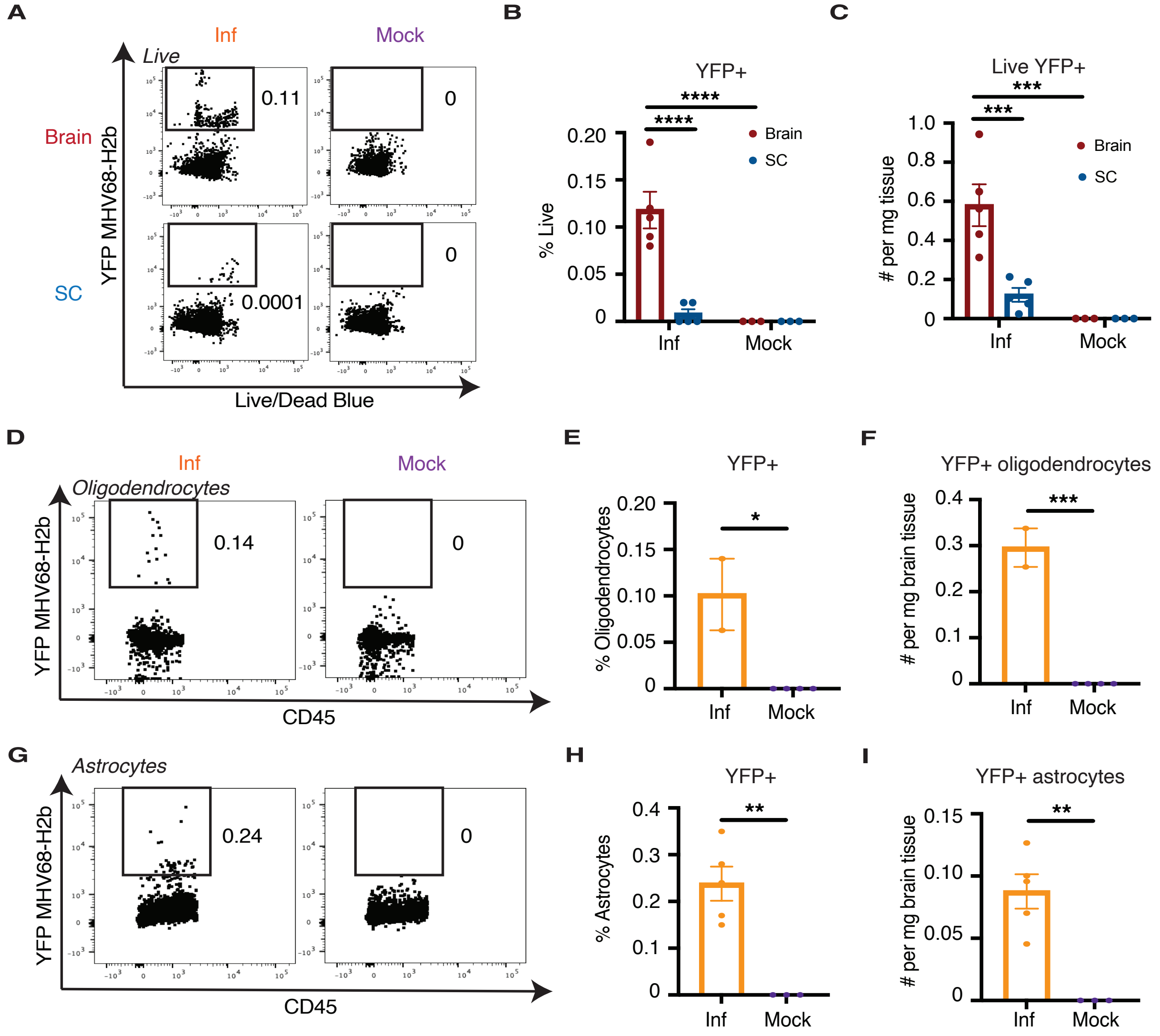

Fig. S1. Intracerebral MHV68 also persists in glial cells residing in the brain, not the spinal cord. (**A**) Brains and spinal cords were isolated on day 32 after intracerebral MHV68-H2bYFP (Inf, n=5) or mock (n=3) infection, and the (**B**) frequency and (**C**) absolute number of live MHV68-H2bYFP+ cells were determined. (**D**) In the brain, the (**E**) frequency and (**F**) absolute number of MHV68-H2bYFP+ oligodendrocytes on day 32 after intracerebral MHV68-H2bYFP (Inf, n=2) or mock (n=4) infection were determined. (**G**) In the brain, the (**H**) frequency and (**I**) absolute number of MHV68-H2bYFP+ astrocytes on day 32 after intracerebral MHV68-H2bYFP (Inf, n=5) or mock (n=3) infection were determined. Statistical significance was determined using a two-way ANOVA with Šidák multiple comparisons in **B** and **C**, and an unpaired 2-tailed t test in **E**, **F**, **H**, and **I**. Data are represented as mean ± standard error of mean (SEM). * $p < 0.05$, ** $p < 0.01$, *** $p < 0.001$, and **** $p < 0.0001$.

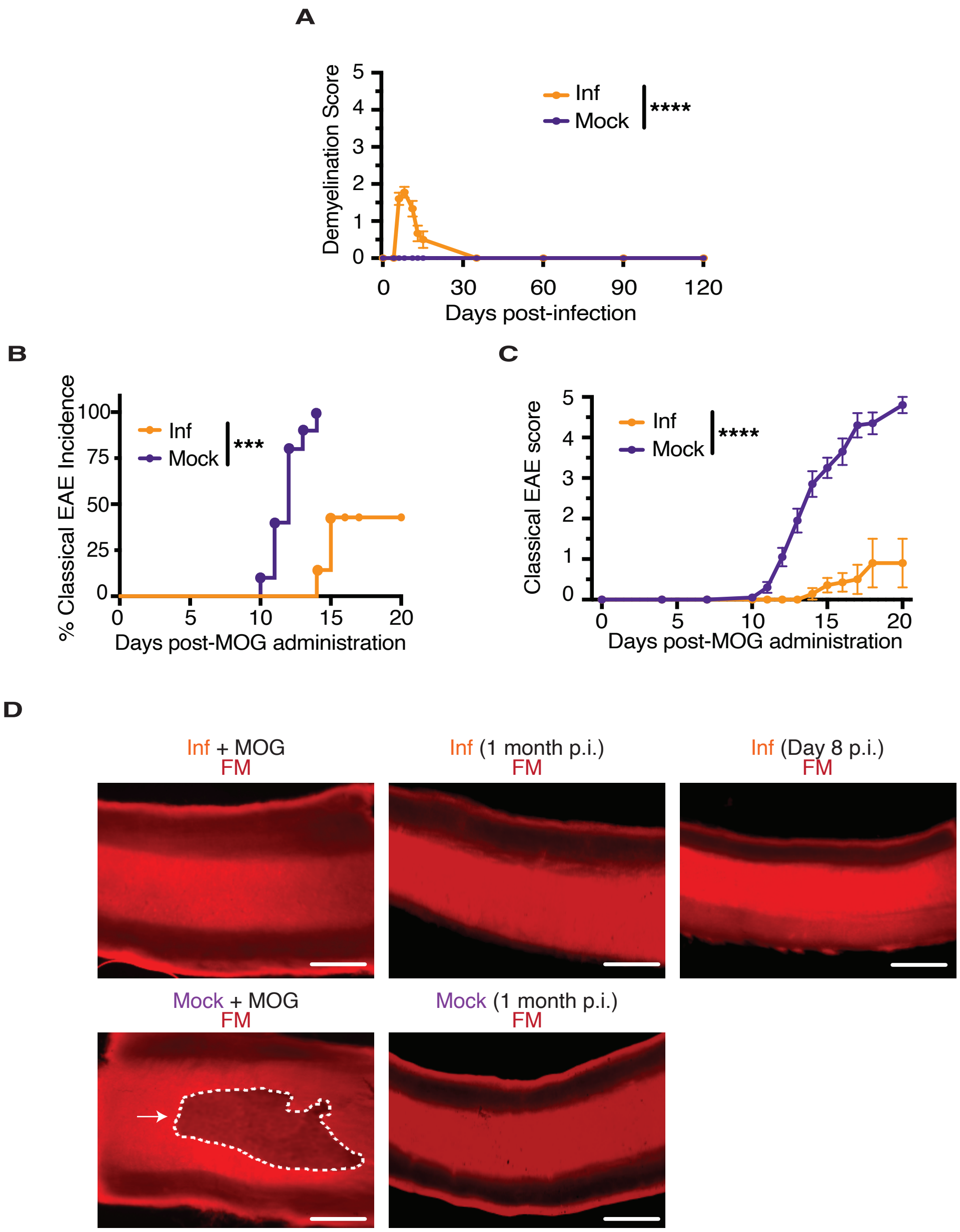

Fig. S2. Mice with persistent intracerebral MHV68 infection exhibit milder spinal cord demyelination following MOG peptide administration. (**A**) Demyelination score, based on TMEV-IDD scoring, following intracerebral MHV68 (Inf, n=10) or mock (n=4) infection. (**B**) Incidence and (**C**) severity of classical EAE symptoms following MOG peptide administration in mice with persistent intracerebral MHV68 infection (Inf, n=7) or mock-infected (n=10). (**D**) Spinal cords were isolated in MHV68-infected (Inf) or mock-infected mice one month or 8 days after infection (p.i.) or 15 days after MOG peptide administration. Fixed frozen sections were stained with FluoroMyelin Red (FM) to assess demyelination. Demyelinated lesion borders are marked with dashed line and arrows. Scale bar, 1000 µm. Statistical significance was determined using a mixed-effects model in **A**-**C**, and a log-rank test in **B**. Data are represented as mean ± standard error of mean (SEM). * $p < 0.05$, ** $p < 0.01$, *** $p < 0.001$, and **** $p < 0.0001$.

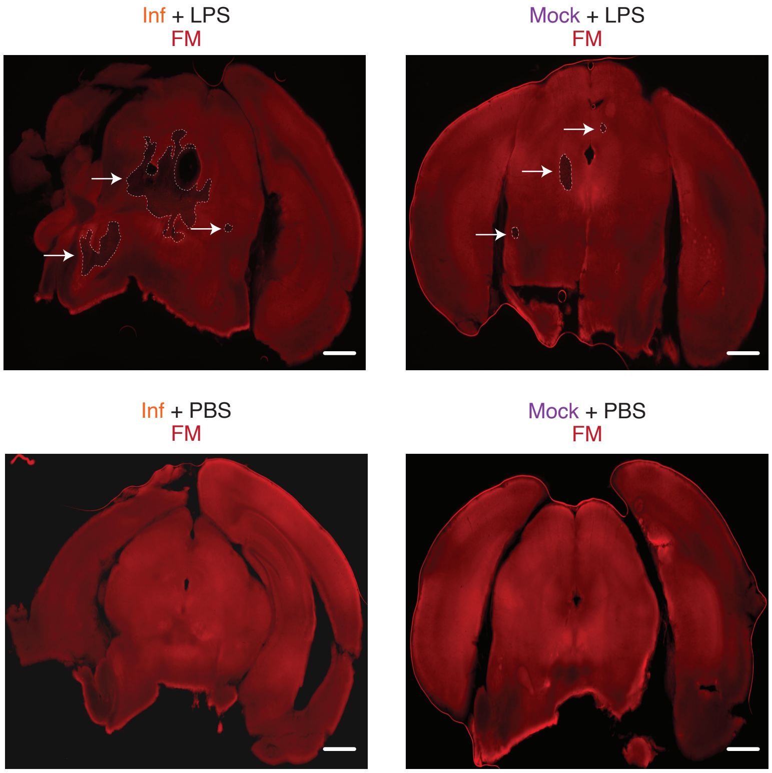

Fig. S3. Persistent intracerebral MHV68 infection exacerbates LPS-induced focal demyelination. LPS or PBS was intracerebrally injected into the midbrain one month after intracerebral MHV68 (Inf) or mock infection. Brains were collected 7 days post-injection, and fixed frozen sections were stained with FluoroMyelin Red (FM) to assess demyelination. Demyelinated lesion borders are marked with dashed line and arrows. Scale bar, 1000 µm.

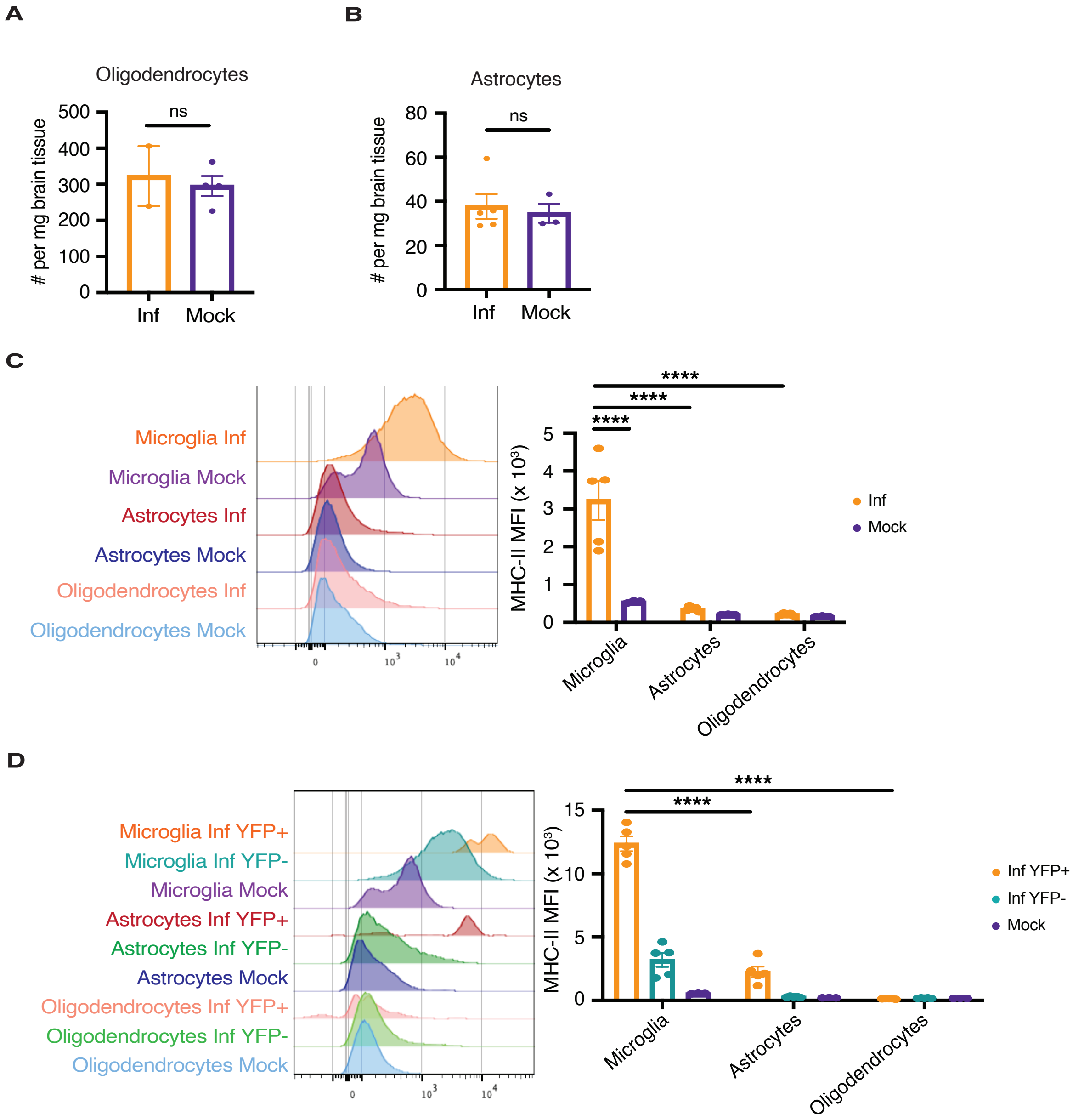

Fig. S4. Oligodendrocytes and astrocytes show minimal changes in abundance and MHC-II expression following intracerebral MHV68 infection. (**A**) Absolute number of oligodendrocytes on day 30 following intracerebral MHV68 (Inf, n=2) or mock (n=4) infection. (**B**) Absolute number of astrocytes on day 30 following intracerebral MHV68 (Inf, n=5) or mock (n=3) infection. (**C**) Comparison of MHC-II expression in microglia, astrocytes, and oligodendrocytes on day 30 following intracerebral MHV68 or mock infection. (**D**) Comparison of MHC-II expression in YFP+ and YFP- microglia, astrocytes, and oligodendrocytes on day 30 following intracerebral MHV68H2bYFP or mock infection. Statistical significance was determined using an unpaired 2-tailed t test in **A** and **B**, and a two-way ANOVA with Šidák correction for multiple comparisons in **C** and **D**. Data are represented as mean ± standard error of mean (SEM). * $p < 0.05$, ** $p < 0.01$, *** $p < 0.001$, and **** $p < 0.0001$.

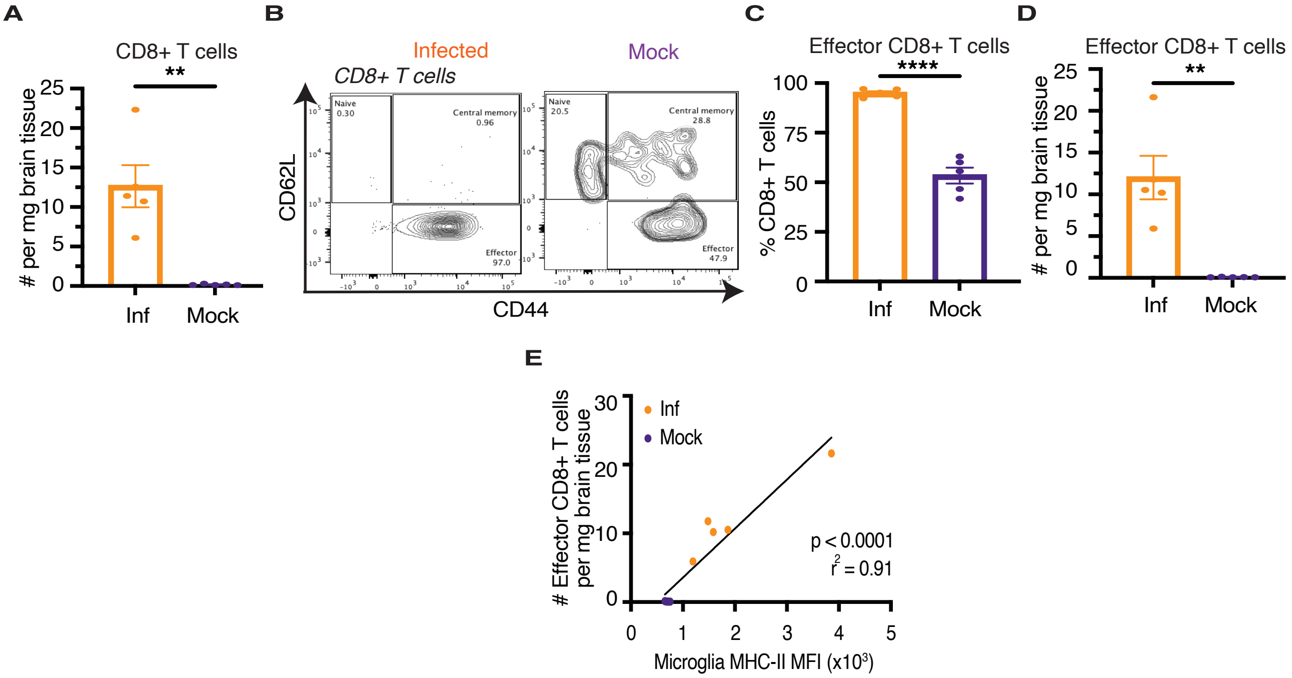

Fig. S5. Persistent intracerebral MHV68 infection induces infiltration and activation of CD8+ T cells into the brain. (**A**) Absolute number of brain-infiltrating CD8+ T cells in mice on day 30 following intracerebral MHV68 (Inf, n=5) or mock infection (n=5). (**B**) On day 30 following intracerebral MHV68 (Inf, n=5) or mock infection (n=5), the (**C**) frequency and (**D**) absolute number of brain-infiltrating effector CD8+ T cells were determined by flow cytometry. (**E**) Correlation between microglial MHC-II expression and the absolute number of brain-infiltrating effector CD8+ T cells on day 30 following intracerebral MHV68 (Inf) or mock infection. Statistical significance was determined using an unpaired 2-tailed t test in **A**, **C**, and **D**, and a simple linear regression in **E**. Data are represented as mean ± standard error of mean (SEM). * $p < 0.05$, ** $p < 0.01$, *** $p < 0.001$, and **** $p < 0.0001$.

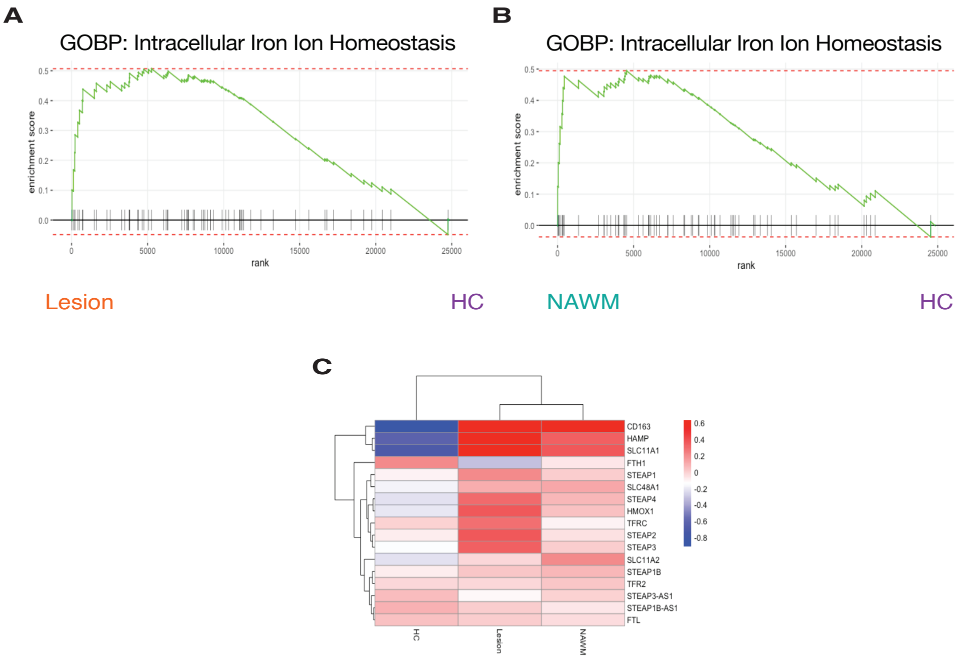

Fig. S6. Altered iron metabolism in microglia residing in MS lesions and NAWM. Gene set enrichment analysis of microglia in (**A**) MS lesions vs healthy control (HC) or (**B**) MS NAWM vs HC brain tissue for the "GOBP: Intracellular Iron Ion Homeostasis" gene set. (**C**) Relative expression of various iron metabolism genes in MS lesions (n=11) and NAWM (n=31) and HC (n=26) brain tissue.

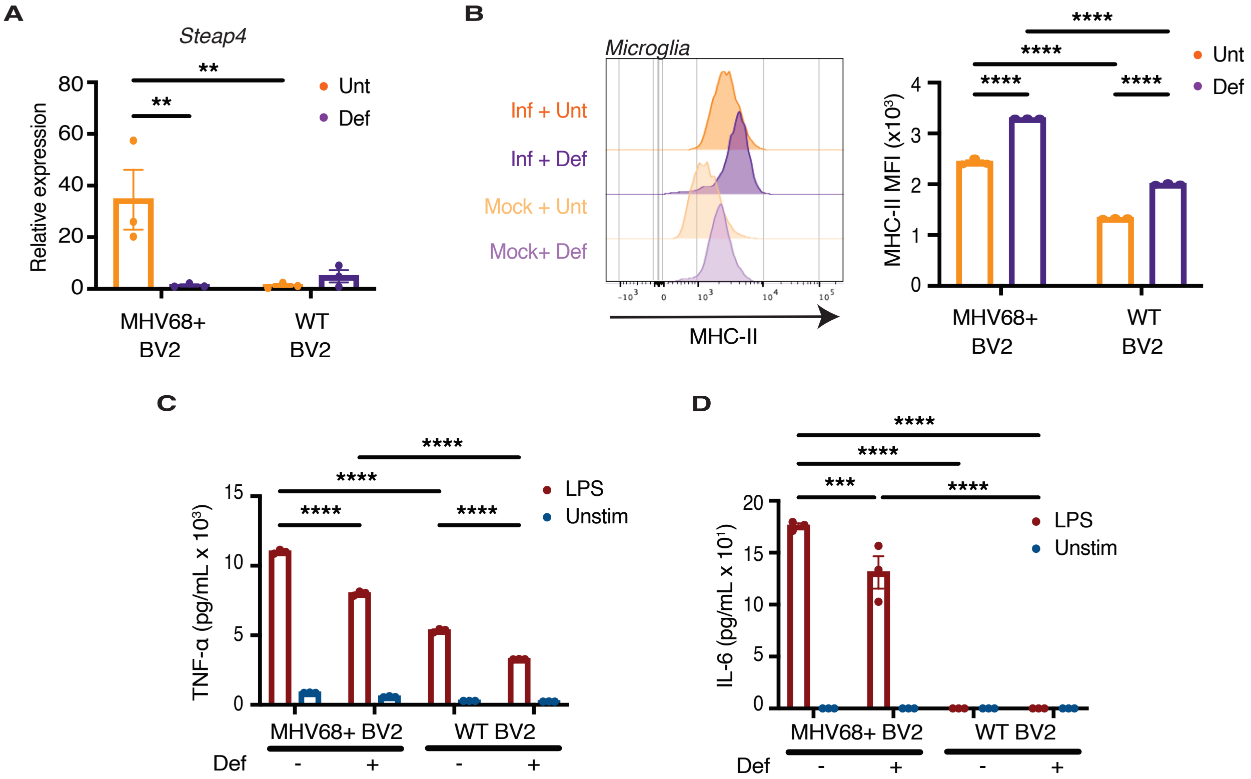

Fig. S7. Iron chelation dampens pro-inflammatory cytokine production in MHV68+ BV2 cells. (**A**) Relative gene expression of Steap4 in MHV68+ BV2 and uninfected (WT) BV2 cells after treatment with 500 µM deferiprone (Def, n=3 technical replicates) or PBS (Unt, n=3 technical replicates). (**B**) MHC-II expression on MHV68+ BV2 and uninfected (WT) BV2 cells treated with 500 µM deferiprone (Def, n=3 technical replicates) or PBS (Unt, n=3 technical replicates). Concentrations of TNF-α (**C**) and IL-6 (**D**) secreted by MHV68+ BV2 and uninfected (WT) BV2 cells upon stimulation with LPS in the presence (Def, n=3 technical replicates) or absence (Unt, n=3 technical replicates) of deferiprone. Statistical significance was determined using a two-way ANOVA with Šidák correction for multiple comparisons in **A**-**D**. Data are represented as mean ± standard error of mean (SEM). * $p < 0.05$, ** $p < 0.01$, *** $p < 0.001$, and **** $p < 0.0001$.

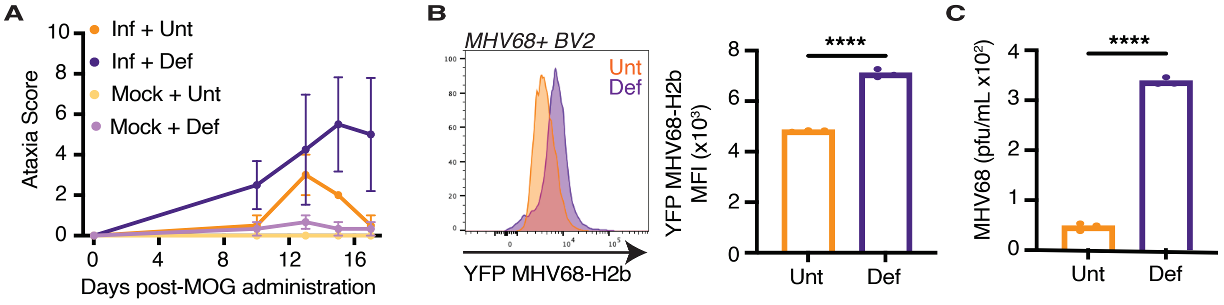

Fig. S8. Iron chelation aggravates MOG peptide-induced ataxia through viral reactivation. (**A**) Mice were intracerebrally infected with MHV68 (Inf, n=7) or mock-infected (n=6) and pre-treated with 75 mg/kg deferiprone (Def, n=7) or PBS (Unt, n=6) daily for 7 days starting one month post-infection. After 7 days of pre-treatment, MOG peptide was administered and treatment was continued throughout EAE development. The severity of ataxia following MOG peptide administration in deferiprone-treated (Def, n=7) or untreated (Unt, n=4) mice after intracerebral MHV68 (Inf, n=6) or mock infection (n=5). (**B**) The expression of MHV68-H2bYFP in MHV68+ BV2 cells treated with 500 µM deferiprone (Def) or PBS (Unt) (n=3 technical replicates per group). (**C**) The concentration of MHV68 in the supernatant of MHV68+ BV2 cells treated with 500 µM deferiprone (Def) or PBS (Unt) (n=3 technical replicates per group). Statistical significance was determined using an unpaired 2-tailed t test in **B** and **C**. Data are represented as mean ± standard error of mean (SEM). * $p < 0.05$, ** $p < 0.01$, *** $p < 0.001$, and **** $p < 0.0001$.

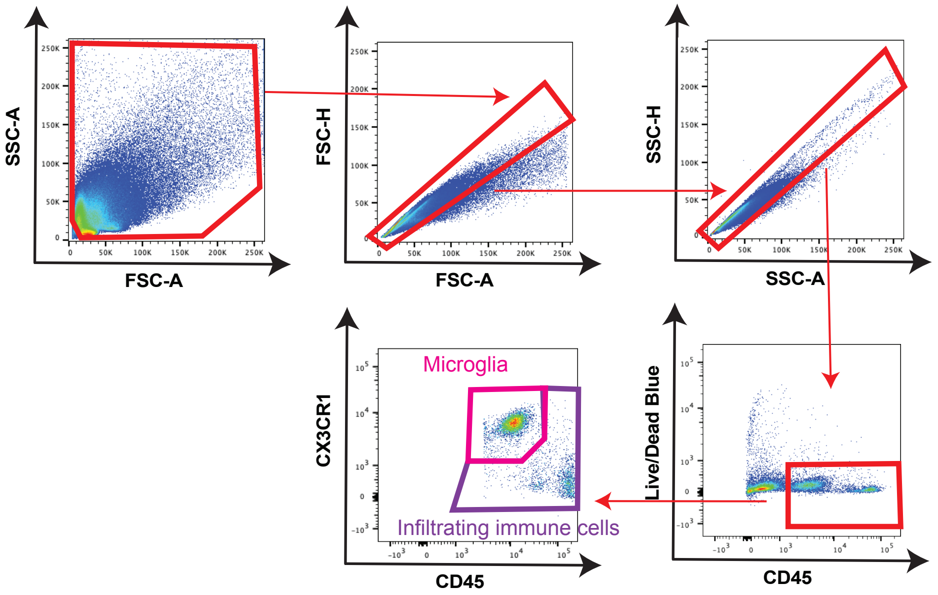

Fig. S9. Flow gating strategy for myeloid cell panel.

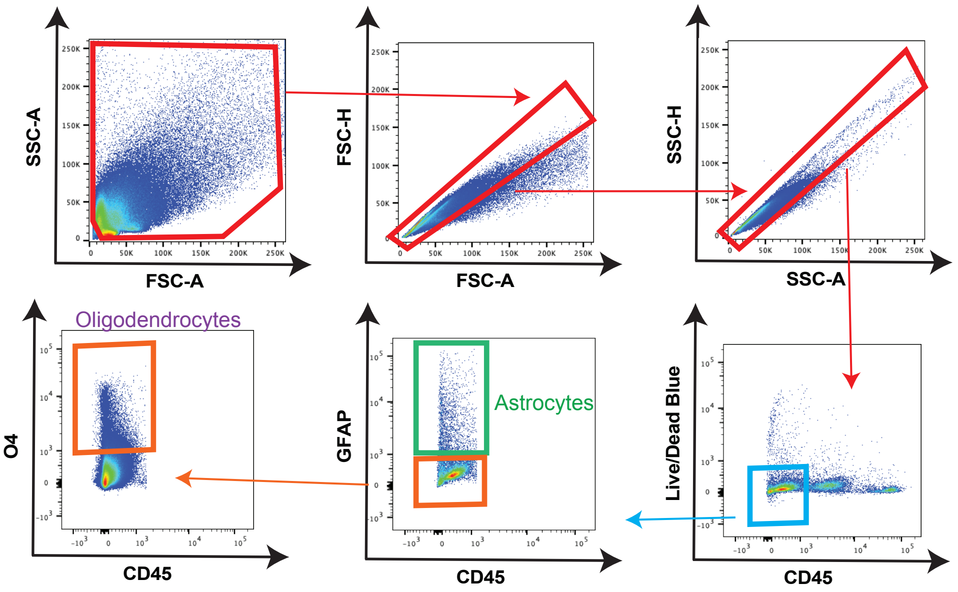

Fig. S10. Flow gating strategy for glial cell panel.

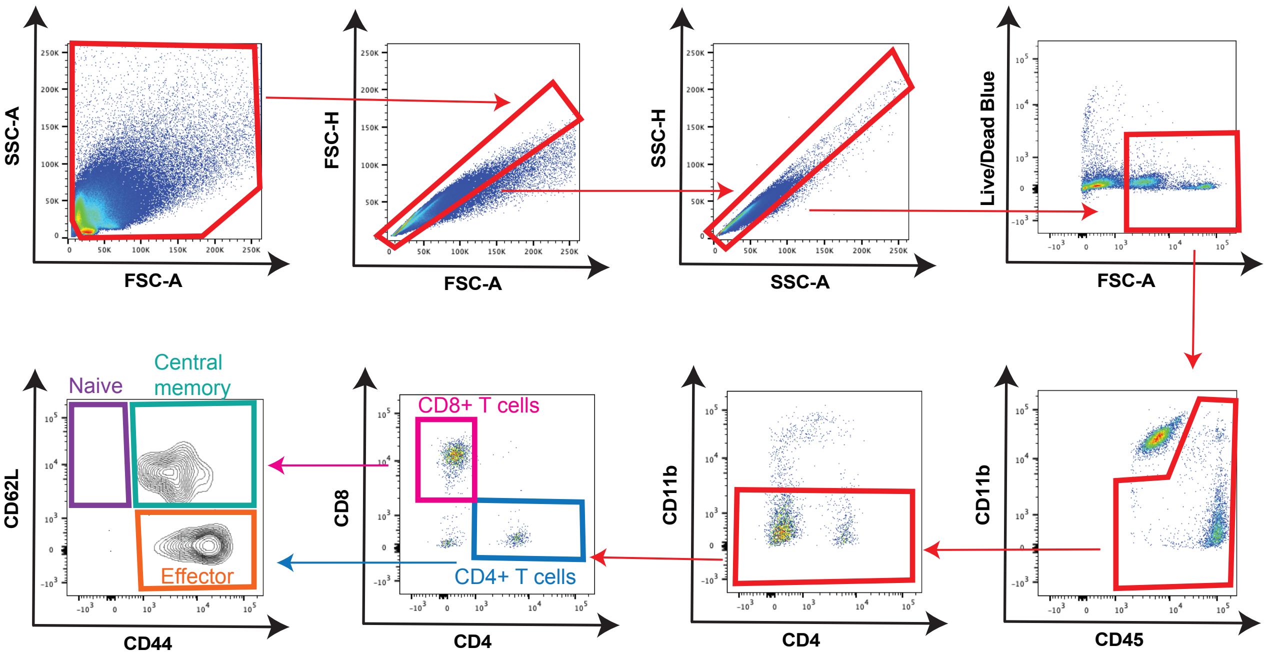

Fig. S11. Flow gating strategy for T cell panel.

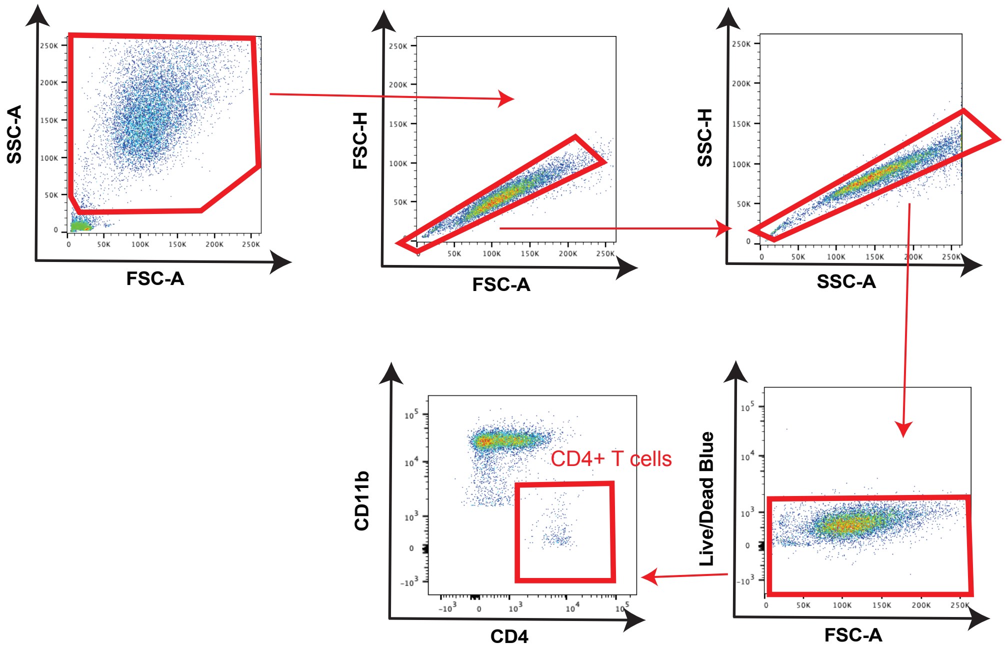

Fig. S12. Flow gating strategy for co-culture panel.

Panel(s)	Target	Channel	Manufacturer	Clone
All	CD45	PE-Cy7	BioLegend	30-F11
All	CD11b	BV421	BioLegend	M1/70
Myeloid	CX3CR1	FITC	BioLegend	SA011F11
Myeloid	I-A/I-E (MHC-II)	BV605	BioLegend	M5/114.15.2
T cell, Co-culture	CD4	PerCP-Cy5.5	Tonbo	RM4-5
T cell	CD8	BV510	BioLegend	53-6.7
T cell	CD44	APC	BioLegend	IM7
T cell	CD62L	PE	BioLegend	MEL-14
Myeloid	TNFα	PE	BioLegend	MP6-XT22
Co-culture	Ki-67	PE	BioLegend	16A8
Co-culture	IFNγ	BV605	BioLegend	XMG1.2
Co-culture	IL-17	AF488	BioLegend	TC11-18H10.1
Glial	GFAP	AF488	BD	1B4
Glial	O4	APC	Miltenyi Biotec	O4

Table S1. Antibodies for flow cytometry.

Gene	Genbank no.	Forward primer (5'-3')	Reverse primer (5'-3')
Slc48a1	NM_026353	GCCATCACCCAGCATCAGAG	GGTGGGCGTAGAGGCTAAGT
Hmox1	NM_010442	GCCACCAAGGAGGTACACAT	AGGAAGCCATCACCAGCTTA
Aco1	NM_007386	CCTGTCCCAGTTAGGGTTTGA	CCAACAGCTACGAGGTCTCC
Aco2	NM_080633	CCTGGGAGGCATCTGCATT	GCTTCACACCGATCACCTTGG
Tfrc	NM_011638	GAAGTCCAGTGTGGGAACAGGT	CAACCACTCAGTGGCACCAACA
Fth1	NM_010239	AGCTGGCATGGCAGAATATC	CTGCCTCAGTGACCAGTAAAG
Lcytb	NM_001362430	CGAGAATCGCACACCTCTACTC	TTTCAGGAGGCTTCGCAGCCAC
Steap2	NM_001285469	GAGCAACGCTTTGAACTGGAGAG	GGCAAGAACGAAGTTTGGTGGTG
Steap3	NM_001085409	TCTTCAGCACCGCCAGTCTAAC	CTGGCTGATCACTGCAGATGAG
Steap4	NM_054098	GGGAATCACTTCCTTGCCATCAG	TCCGCCATACACCAAAGTGTGG
Dmt1	NM_001146161	TTGCAGCGAGACTTGGAGTGGT	GCTGAGCCAATGACTTCCTGCA
Fpn	NM_016917	CCATAGTCTCTGTCAGCCTGCT	CTTGCAGCAACTGTGTCACCGT
Gapdh	NM_001289726	CATCACTGCCACCCAGAAGACTG	ATGCCAGTGAGCTTCCCGTTCAG

Table S2. Sequences of qPCR primer pairs.
